# Image-based artificial intelligence for preoperative differentiation of pancreatic cancer from pancreatitis: a systematic review and meta-analysis

**DOI:** 10.3389/fonc.2025.1660271

**Published:** 2026-01-12

**Authors:** Juan Lu, Haiyi Zhang, Zhengzhen Yuan, Jiajun Yue, Qi Yao, Yong Liu, Pingping Jie, Min Fan, Jie Zhao

**Affiliations:** 1 Department of Magnetic Resonance Imaging, The Affiliated Traditional Chinese Medicine Hospital, Southwest Medical University, Luzhou, China; 2 School of Physical Education, Southwest Medical University, Luzhou, Sichuan, China; 3 The Affiliated Traditional Chinese Medicine Hospital, Southwest Medical University, Luzhou, China; 4 Department of Nuclear Medicine, The Affiliated Hospital of Southwest Medical University, Luzhou, China; 5 Department of Nuclear Medicine and Molecular Imaging Key Laboratory of Sichuan Province, The Affiliated Hospital of Southwest Medical University, Luzhou, China

**Keywords:** artificial intelligence, preoperative diagnosis, pancreatic cancer, pancreatitis, differential diagnosis, meta-analysis

## Abstract

**Background:**

Pancreatic cancer (PC) and pancreatitis—encompassing acute, chronic, autoimmune, and other inflammatory pancreatic conditions—often exhibit overlapping clinical and imaging features, yet require fundamentally different therapeutic strategies. This similarity frequently leads to diagnostic uncertainty in routine clinical practice. Image-based artificial intelligence (AI) has emerged as a promising tool to enhance diagnostic accuracy. This meta-analysis systematically evaluates the diagnostic performance of AI algorithms in differentiating PC from pancreatitis.

**Methods:**

A systematic literature search of PubMed, Embase, and Cochrane Library databases was conducted for studies published through June 30 2025. Eligible studies reporting AI diagnostic performance metrics were selected. Methodological rigor was assessed using the modified Quality Assessment of Diagnostic Accuracy Studies (QUADAS-2) tool. Pooled sensitivity (SEN), specificity (SPE), positive/negative likelihood ratios (+LR/-LR), diagnostic odds ratio (DOR), and summary receiver operating characteristic (SROC) curves were derived using Stata 17.0 software.

**Results:**

Twenty-five eligible studies (3279 patients) were ultimately eligible for data extraction, of which sixty-eight tables were included in this meta-analysis. The pooled SEN was 89% (95% CI: 87–90%), SPE was 88% (95% CI: 86–90%), and AUC was 0.94 (95% CI: 0.92–0.96) in 28 included studies with 76 contingency tables, however, substantial heterogeneity was observed among the included studies, with I² = 77.14% in SEN and I² = 75.61% in SPE. The pooled SEN and SPE were 91% (95% CI: 88–93%) and 90% (95% CI: 87–93%), with an AUC of 0.96 (95% CI: 0.94–0.97) in 28 included studies with 28 best diagnosis performance tables. Analysis for different algorithms revealed a pooled SEN of 89% (95%CI: 86−90%) and SPE of 88% (95%CI: 86−90%) for machine learning, and a pooled SEN of 89% (95%CI: 82−93%) and SPE of 85% (95%CI: 76−91%) for deep learning. Subsequent subgroup analysis suggested that part of the heterogeneity might be explained by differences in Algorithm, Imaging Modality, Publication Geographical, and Year of publication.

**Conclusion:**

AI-based image analysis demonstrates strong diagnostic performance in distinguishing PC from pancreatitis, exceeding thresholds typically achieved with conventional imaging alone. These findings support the potential integration of AI into clinical decision-support workflows to improve the preoperative evaluation of pancreatic lesions.

**Systematic review registration:**

https://www.crd.york.ac.uk/prospero/, identifier CRD42024529580.

## 1 Introduction

Pancreatic cancer (PC), characterized by its aggressive biological behavior, rapid progression, and dismal prognosis, remains one of the most lethal malignancies worldwide. While surgical resection represents the sole curative option for patients with PC (1, 2), its clinical management is complicated by the diagnostic challenge of distinguishing it from pancreatitis such as mass-forming pancreatitis (MFP), mass-forming chronic pancreatitis (MFCP), autoimmune pancreatitis (AIP), focal forms of chronic pancreatitis, and other focal inflammatory lesions. These two entities share overlapping clinical presentations (including weight loss, abdominal pain, and pancreatic insufficiency) and imaging features (3, 4), yet demand diametrically opposed treatment strategies: pancreatitis typically responds to medical therapy whereas PC requires radical resection. Accurate preoperative differentiation is therefore critical to avoid two detrimental scenarios—delayed intervention in early-stage PC (potentially compromising survival outcomes) and unnecessary pancreatic surgery in pancreatitis cases (5–8).

Imaging diagnosis plays a pivotal role in this differential diagnostic process (9, 10). Conventional radiographic evaluation relies heavily on qualitative assessment of morphological features, yet modern imaging techniques [such as computed tomography (CT), magnetic resonance imaging (MRI), and positron emission tomography (PET)] generate vast amounts of quantitative data that may provide greater diagnostic value than traditional imaging biomarkers in diagnostic value (11–14). Current clinical practice, however, remains constrained by interobserver variability and a reliance on radiologists’ subjective experience in evaluating heterogeneous pancreatic lesions (15, 16).

The emergence of artificial intelligence (AI) has introduced transformative potential in oncological imaging. Contemporary research demonstrates AI’s capacity to extract high-throughput quantitative features from medical images, including subvisual patterns imperceptible to human observers—thereby enhancing diagnostic precision and prognostic stratification (17–19). Technological advancements in computational power, algorithm optimization, and the availability of large-scale imaging datasets have accelerated AI applications via multimodal data integration (encompassing radiological, histopathological, genomic, and metabolic information) to refine clinical decision-making (20–22). Broadly, image-based AI methods can be categorized into radiomics-based machine learning (ML) and deep learning (DL). Radiomics-based ML requires manual or semi-automatic segmentation of lesions, followed by extraction of handcrafted quantitative features and classification using algorithms such as support vector machines (SVMs), random forests (RFs), or LASSO regression. By contrast, DL methods such as convolutional neural networks (CNNs) or deep learning radiomics (DLR) automatically learn and optimize image features in an end-to-end manner with minimal manual input. In our systematic review, the majority of included studies adopted radiomics-based ML approaches, whereas a smaller subset used DL architectures. This context provides an essential framework for interpreting the pooled diagnostic performance reported in the following sections. Notably, evidence from multiple comparative studies indicates that AI systems can perform at least comparably to radiologists, with some reports showing similar or occasionally superior accuracy in differentiating PC from pancreatitis across different imaging modalities (23). Nevertheless, a comprehensive quantitative synthesis of this evidence remains lacking (24, 25).

In this study, we conducted a comprehensive review and meta-analysis of published diagnostic data in order to better understand how well AI algorithms and models perform in the differential diagnosis of PC from pancreatitis and explored the clinical applicability.

## 2 Materials and methods

This systematic review was performed according to the Preferred Reporting Items for Systematic Reviews and Meta-analysis for Diagnostic Test Accuracy (PRISMA-DTA) guidelines (26). The protocol of this meta-analysis was registered and is available at PROSPERO (No. CRD42024529580). Literature search, data extraction, and quality assessment were performed independently by two reviewers (H.Y. Zhang and J. Lu).

### 2.1 Literature search and selection

PubMed, Embase, and the Cochrane Library databases were systematically searched for studies published until 30 June 2025. The search was limited to English-language publications. A detailed search strategy was tailored for each database, using a combination of Medical Subject Headings (MeSH) terms and free-text keywords. The search strategy included the following subject headings and abstract words: artificial intelligence, radiomics, deep learning, machine learning, pancreas, pancreatic cancer, pancreatic adenocarcinoma, mass-forming pancreatitis, autoimmune pancreatitis, and focal chronic pancreatitis. The search strategy was: (“radiomics” OR “deep learning” OR “machine learning” OR “artificial intelligence”) AND (“pancreatic cancer” OR “pancreatic adenocarcinoma”) AND (“mass-forming pancreatitis” OR “autoimmune pancreatitis” OR “focal chronic pancreatitis”). Boolean operators (“AND”, “OR”) and proximity operators were used to broaden the search and capture variations in terminology. The complete electronic search strategies for all databases, including MeSH terms, keywords, Boolean operators, and date limits, are provided in **Supplementary Table S1** for transparency and reproducibility. The strategy was adjusted to accommodate each database’s specific indexing systems and syntaxes. Following deduplication, two investigators independently screened titles and abstracts. Articles deemed potentially relevant underwent full-text review against predefined inclusion criteria. Studies satisfying these criteria were incorporated into qualitative synthesis and meta-analyses. Additionally, backward citation tracking of included articles and related systematic reviews was performed to identify supplementary evidence. Throughout the screening procedure, the corresponding author was consulted in order to settle any disputes among the authors on research selection.

### 2.2 Inclusion criteria

The inclusion criteria were designed based on the following PICOS criteria to ensure comprehensive and unbiased searches as follows:

- P (Population) and S (Study): patients with PC [including pancreatic cancer (PC) or pancreatic ductal adenocarcinoma (PDAC)] or pancreatitis [including mass-forming pancreatitis (MFP), chronic pancreatitis (CP), autoimmune pancreatitis (AIP), and mass-forming chronic pancreatitis (MFCP), and other focal inflammatory lesions] confirmed by histological or clinical diagnoses.
- I (Intervention): AI-based diagnostic systems (e.g., ML models and DL algorithms) designed to differentiate PC from pancreatitis.
- C (Comparator): Histopathological confirmation (biopsy/surgical specimen) or comprehensive clinical diagnosis (imaging follow-up ≥ 12 months with multidisciplinary consensus) results were used as the reference standard to compare the performance of AI algorithms.
- O (Outcomes): Diagnostic accuracy for differentiating pancreatitis from PC. The performance of AI algorithms was assessed through key metrics, including the area under the curve (AUC), sensitivity, and specificity data of the models or the corresponding information for constructing a 2 × 2 matrix table.

Exclusion criteria were as follows: (a) studies involving patients who had received neoadjuvant therapy (including immunotherapy, radiotherapy, or chemotherapy) prior to diagnostic imaging; (b) studies failing to provide extractable data for reconstructing 2×2 contingency tables or explicit diagnostic validation protocols; (c) non-original research publications (case reports, systematic reviews, editorials, and letters) and conference abstracts without full methodological details; and (d) investigations exclusively focusing on technical validation phases such as image segmentation algorithms or radiomic feature extraction pipelines.

### 2.3 Data extraction and quality assessment

Two reviewers (H.Y. Zhang and J. Lu) independently extracted the data from the included studies. The following data were extracted from the eligible studies: (a) study characteristics (the first author, year of publication, country, and study design); (b) lesion characteristics and numbers of PC or pancreatitis; (c) AI algorithms and subtype (DL or ML); (d) images modalities (CT, MRI, ECT, and PET/CT); (e) the reference criteria for pancreatitis and PDAC (histopathological or clinical diagnoses); and (f) the diagnostic accuracy data including true-positive (TP), false-positive (FP), true-negative (TN), and false-negative (FN) were extracted directly into contingency tables, which were used to calculate SE and SP. If a study provided multiple contingency tables for the same or different AI algorithms, we assumed that they were independent of each other.

The quality of the reviewed studies was evaluated using the Quality Assessment of Diagnostic Accuracy Studies 2 (QUADAS-2) criteria. The tool assesses four key domains: “Patient Selection”, “Index Test”, “Reference Standard”, and “Flow and Timing”. For each domain, two reviewers independently evaluated the risk of bias (high/low/unclear) and applicability concerns (high/low). Discrepancies were resolved through discussion or consultation with a third researcher.

### 2.4 Meta-analysis

Statistical analyses were conducted by using STATA 17.0 (StataCorp LLC, College Station, Texas, USA) using the bivariate random-effects model. Coupled forest plots were generated to visually present pooled sensitivity and specificity. The pooled sensitivity and specificity of the differential diagnosis of pancreatitis and PC, and their 95% confidence intervals (CIs) were calculated using the relevant data extracted from each individual study. The sensitivity was calculated by dividing the number of patients who had a PC diagnosis by the total number of patients with PC, and the specificity was calculated by dividing the number of patients who had an MPF by the total number of MPF patients. *I*^2^ values were computed to assess statistical heterogeneity, categorized as very low (0%–25%), low (25%–50%), medium (50%–75%), and high (>75%) [19,20]. Publication bias was determined by visual assessment of Deeks’ funnel plot, and statistical significance was evaluated by Deeks’ asymmetry test. *p* < 0.05 was considered statistically significant.

If significant heterogeneity presented, a subgroup analysis was conducted to investigate the cause of heterogeneity. The following covariates were used in the subgroup analysis: (a) subtype of AI algorithms (ML *vs*. DL); (b) imaging modalities (US, CT, MRI, or PET); (c) according to the pooled performance using the same dataset (AI algorithms or human clinicians); (d) according to the geographical distribution (Asia or non-Asia); (e) number of institutions from which data were obtained (single or multiple centers); (f) patient numbers (patients ≤100 *vs*. patients >100); (g) publication year (before 2020 *vs*. after 2020); (h) studies were categorized as low risk or high/unclear risk of bias, based on the overall judgment across the four domains (patient selection, index test, reference standard, and flow/timing); and (i) different AI algorithm (LASSO *vs*. SVM *vs*. ANNs).

### 2.5 Sensitive analysis

A sensitivity analysis was performed by excluding studies with high or unclear risk of bias to evaluate the influence of lower-quality studies on the overall pooled results. The pooled sensitivity, specificity, diagnostic odds ratio (DOR), and area under the summary receiver operating characteristic curve (AUC) were recalculated for the remaining studies The results were compared with the primary analysis to assess the stability and robustness of the findings.

## 3 Results

### 3.1 Study selection and characteristics of eligible studies

**Figure 1:**
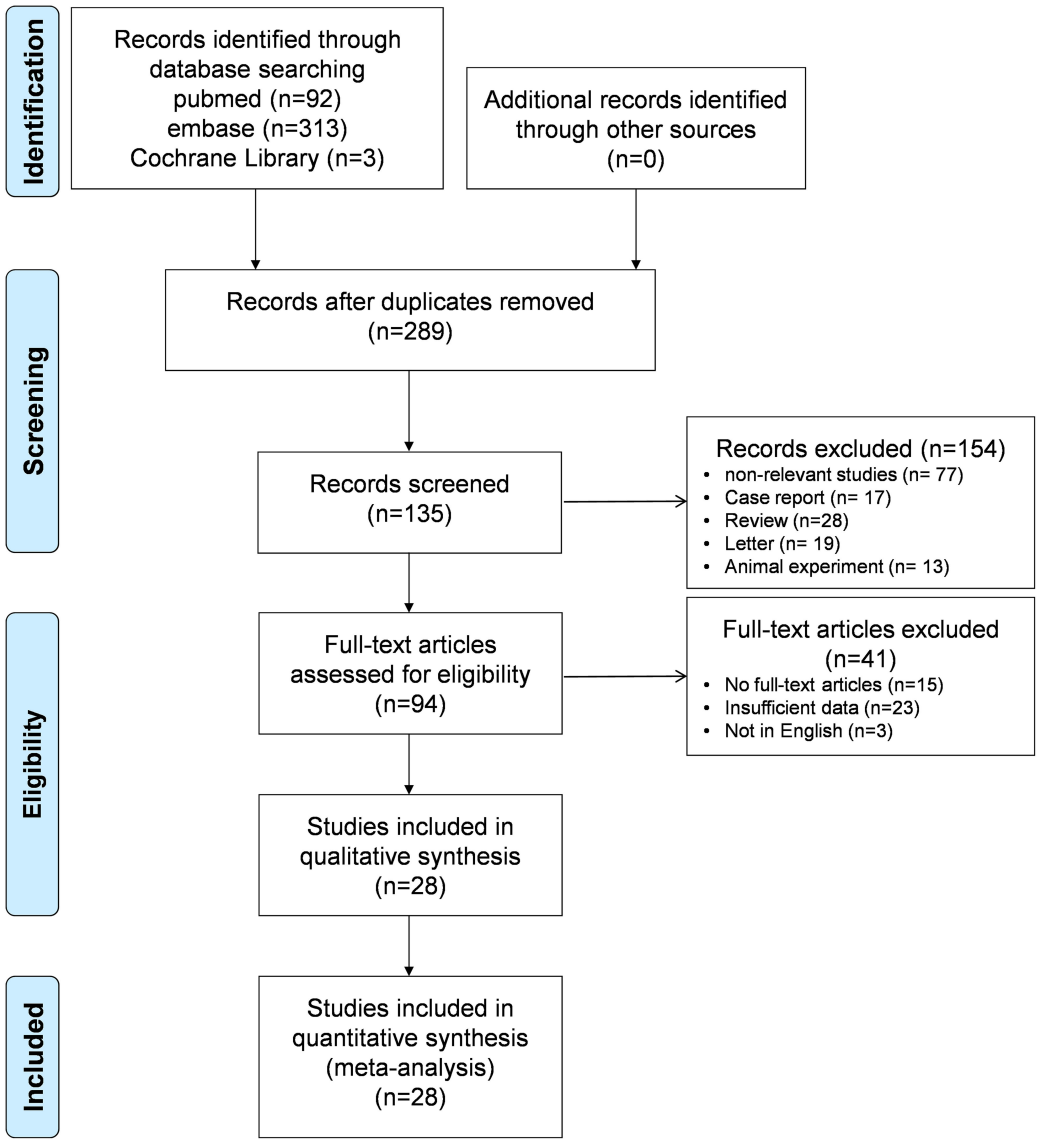
Flowchart of the selection of studies.

A total of 408 studies were retrieved during the initial search. After removing 119 duplicates, 135 studies were excluded based on title and abstract screening, leaving 94 studies eligible for full-text review. Ultimately, 28 articles were included in the systematic review, all of which provided sufficient data for meta-analysis. The final analysis incorporated data from 3,279 patients. A total of 76 contingency tables, including TP, FP, TN, and FN, were extracted from the 28 eligible studies (3, 24, 25, 27–49). The complete details of the literature search and screening process are illustrated in **Figure 1** and **Supplementary Table S1**.

The included studies were published between 2001 and 2025, with a notable concentration of 20 publications (71.4%) between 2019 and 2023. The majority of the studies (*n* = 17) were conducted in China, while the remaining studies were carried out in America, Japan, Romania, Germany, and India. Among the included studies, 25 employed retrospective designs, and 3 utilized prospective designs. Twenty-two studies were conducted at a single center, while six involved multiple centers. Eight studies compared the performance of AI models with clinicians using the same dataset. Twenty-four studies explicitly excluded low-quality images, whereas the remaining studies did not mention this process.

The imaging modalities used in the studies were categorized as follows: CT (*n* = 11), MRI (*n* = 4), US (*n* = 9), and PET/CT (*n* = 4). Twenty-three studies analyzed retrospectively collected data, while the remaining three studies used prospectively collected data. In terms of AI algorithms, the distribution was as follows: ML (25 studies) and DL (3 studies). Eight studies compared the performance of AI with that of expert clinicians. Detailed characteristics of the included studies are presented in **Tables 1**, **2** and **Supplementary Tables S2**, **S3**.

**Table 1:** Main characteristics of the included studies.

Author	Year	Country	Patients	Study design	Centers	PC/PDAC	Pancreatitis	Reviewers	Blinded
Norton	2001	USA	35	Retro	S	21 PDAC	14 CP	NR	NR
Adrian	2008	Romania	43	Prosp	S	32 PDAC	11 CP	NR	Yes
Adrian	2012	Romania	258	Prosp	M	211 PC	47 CP	NR	Yes
Zhu	2013	China	388	Retro	S	262 PC	126 CP	NR	NR
Adrian	2015	Romania	167	Prosp	M	112 PC	55 CP	NR	Yes
Zhang	2019	China	111	Retro	S	66 PDAC	45 AIP	NR	NR
Ren	2019	China	109	Retro	S	79 PDAC	30 MFP	3	Yes
Park	2020	USA	62	Retro	S	29 PDAC	33 AIP	3	Yes
Lin	2020	China	96	Retro	S	51 PDAC	45 AIP	3	Yes
Marya	2020	USA	438	Retro	S	292 PDAC	146 AIP	NR	NR
Ziegelmayer	2020	Germany	86	Retro	S	42 PDAC	44 AIP	2	NR
Ren	2020	China	109	Retro	S	79 PDAC	30 MFP	NR	NR
Liu	2021	China	112	Retro	S	64 PDAC	48 AIP	NR	NR
Li	2021	China	97	Retro	S	55 PDAC	42 AIP	2	Yes
Deng	2021	China	64	Retro	M	51 PDAC	13 MFCP	2	Yes
Tong	2022	China	109	Retro	M	73 PDAC	36 CP	5	Yes
Zhang	2022	China	111	Retro	S	66 PDAC	45 AIP	2	Yes
Anai	2022	Japan	50	Retro	S	30 PDAC	20 AIP	4	Yes
Wei	2022	China	112	Retro	S	64 PDAC	48 AIP	NR	NR
Shiraishi	2022	Japan	105	Retro	S	77 PDAC	28 AIP	2	Yes
Liu	2022	China	72	Retro	S	38 PC	34 MFCP	2	NR
Ma	2022	China	175	Retro	M	151 PDAC	24 MFCP	2	NR
Zhang	2022	China	35	Retro	S	12 PDAC	23 MFCP	2	Yes
Lu	2023	China	67	Retro	S	45 PDAC	22 AIP	2	Yes
Malagi	2023	India	31	Retro	S	25 PDAC	6 MFCP	2	Yes
Nakamura	2024	Japan	85	Retro	S	61 PDAC	24 AIP	2	Yes
Zhang	2025	China	110	Retro	M	55 PDAC	55 AIP	3	Yes

S: single center; M: multi-center.

Retro: retrospectively; Prosp: prospectively.

PC: pancreatic cancer; PDAC: pancreatic ductal adenocarcinoma.

Pancreatitis: mass-forming pancreatitis; CP: chronic pancreatitis; AIP: autoimmune pancreatitis; MFCP: mass-forming chronic pancreatitis.

**Table 2:** Indicator, algorithm, and data source for the 25 included studies.

Author and year	Imaging modality	Exclusion of poor quality imaging	Heatmap provided	Algorithm classifier	ML/DL	AI versus clinicians
Norton 2001	EUS	NR	NR	ANNs	ML	Yes
Adrian 2008	EUS	Yes	NR	ANNs	ML	No
Adrian 2012	EUS	Yes	NR	ANNs	ML	No
Zhu 2013	EUS	Yes	NR	SVM	ML	No
Adrian 2015	EUS	Yes	NR	ANNs	ML	No
Zhang 2019	PET/CT	NR	NR	SVM	ML	Yes
Ren 2019	CT	Yes	NR	NA	ML	No
Park 2020	CT	Yes	NR	RF	ML	No
Lin 2020	CT	Yes	NR	RF	ML	Yes
Marya 2020	EUS	Yes	NR	CNNs	ML	NR
Ziegelmayer 2020	CT	Yes	NR	CNNs	ML	NR
Ren 2020	CT	NR	NR	RF	ML	NR
Liu 2021	PET/CT	NR	NR	SVM-RFE	ML	NR
Li 2021	CT	Yes	NR	LASSO	ML	NR
Deng 2021	MRI	Yes	NR	SVM	ML	YES
Tong 2022	EUS	Yes	NR	DLR	DL	YES
Zhang 2022	PET/CT	Yes	NR	SVM-RFE	ML	No
Anai 2022	CT	Yes	NR	SVM	ML	No
Wei 2022	PET/CT	Yes	NR	MF	DL	No
Shiraishi 2022	MRI	Yes	NR	SVM	ML	No
Liu 2022	MRI	Yes	NR	LASSO	ML	Yes
Ma 2022	CT	Yes	NR	LASSO	ML	Yes
Zhang 2022	CT	Yes	NR	LASSO	ML	No
Lu 2023	CT	Yes	NR	RF	ML	No
Malagi 2023	MRI	Yes	NR	ANNs	ML	No
Qu 2023	CT	Yes	NR	LASSO	ML	No
Nakamura 2024	EUS	Yes	NR	CNNs	DL	Yes
Zhang 2025	EUS	Yes	NR	LASSO	ML	No

S, single center; M, multi-center.

R, retrospectively; P, prospectively.

ML, machine learning; DL, deep learning; ANNs, artificial neural networks; SVM, support vector machine; NA, not available.

RF, random forest; CNNs, convolutional neural networks; SVM-RFE, support vector machine–recursive feature elimination.

LASSO, least absolute shrinkage and selection operator; DLR, deep learning radiomics; MF, multidomain fusion.

### 3.2 Quality assessment

The quality of included studies was determined by the QUADAS-2 (**Supplementary Figure S1**). The detailed assessment results are presented in **Supplementary Figure S2**. Over half of the studies showed a high risk or an unclear risk of bias respectively for patient selections (*n* = 16) because these studies did not clearly describe patient characteristics, including prior testing, clinical presentation, study setting, intended use of the index test, or external evaluation. The risk of bias in the index test was low in 13 studies (46%) and high or unclear in 15 studies (54%). The risk of bias within the domains of reference standard tests was consistently low across all studies. Moreover, 15 studies remained unclear in the flow and timing domain, while 13 studies were low in this regard. The analysis revealed a potential risk of bias in the domain of patient selection, with the absence of randomization serving as a primary contributing factor. Overall, the included literature was deemed suitable for subsequent analyses in light of its quality.

### 3.3 Pooled performance of AI algorithms

**Figure 2:**
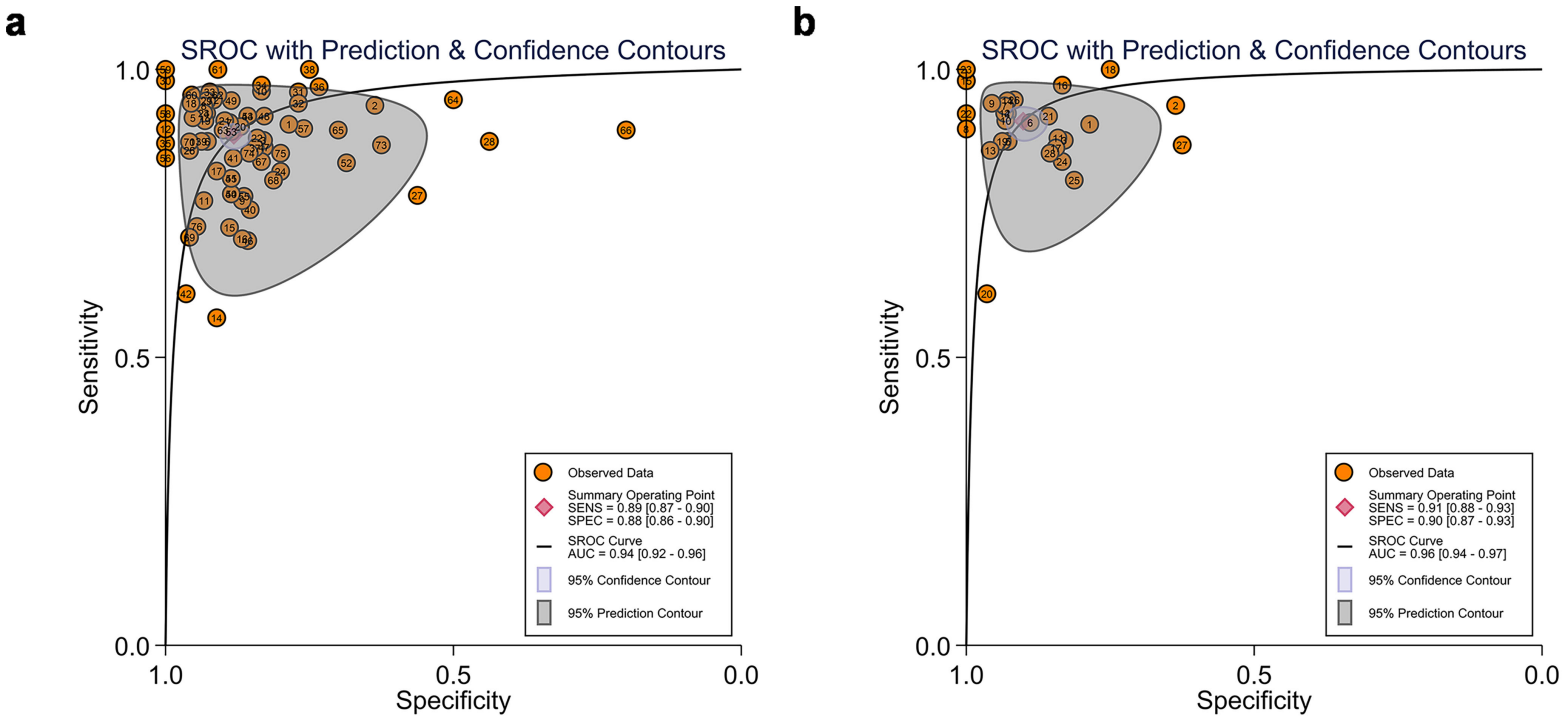
**(a)** Summary receiver operating characteristic (SROC) curve (28 studies with 76 tables). **(b)** SROC curves of studies when selecting contingency tables reporting the highest accuracy (28 studies with 28 best values).

**Figure 3:**
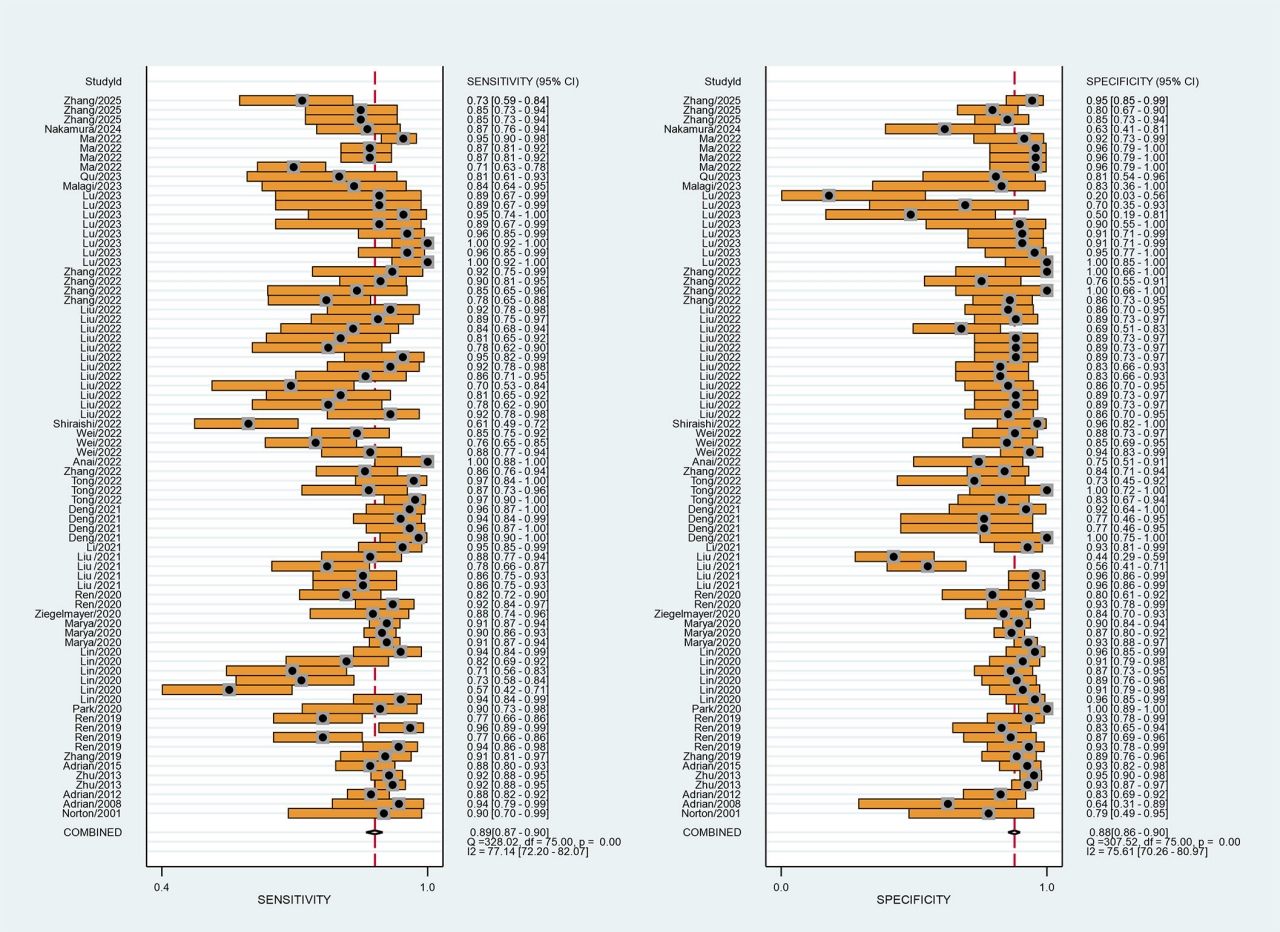
Forest plot of studies when selecting contingency tables reporting the Sen and Spe (28 studies with 76 best values).

**Figure 4:**
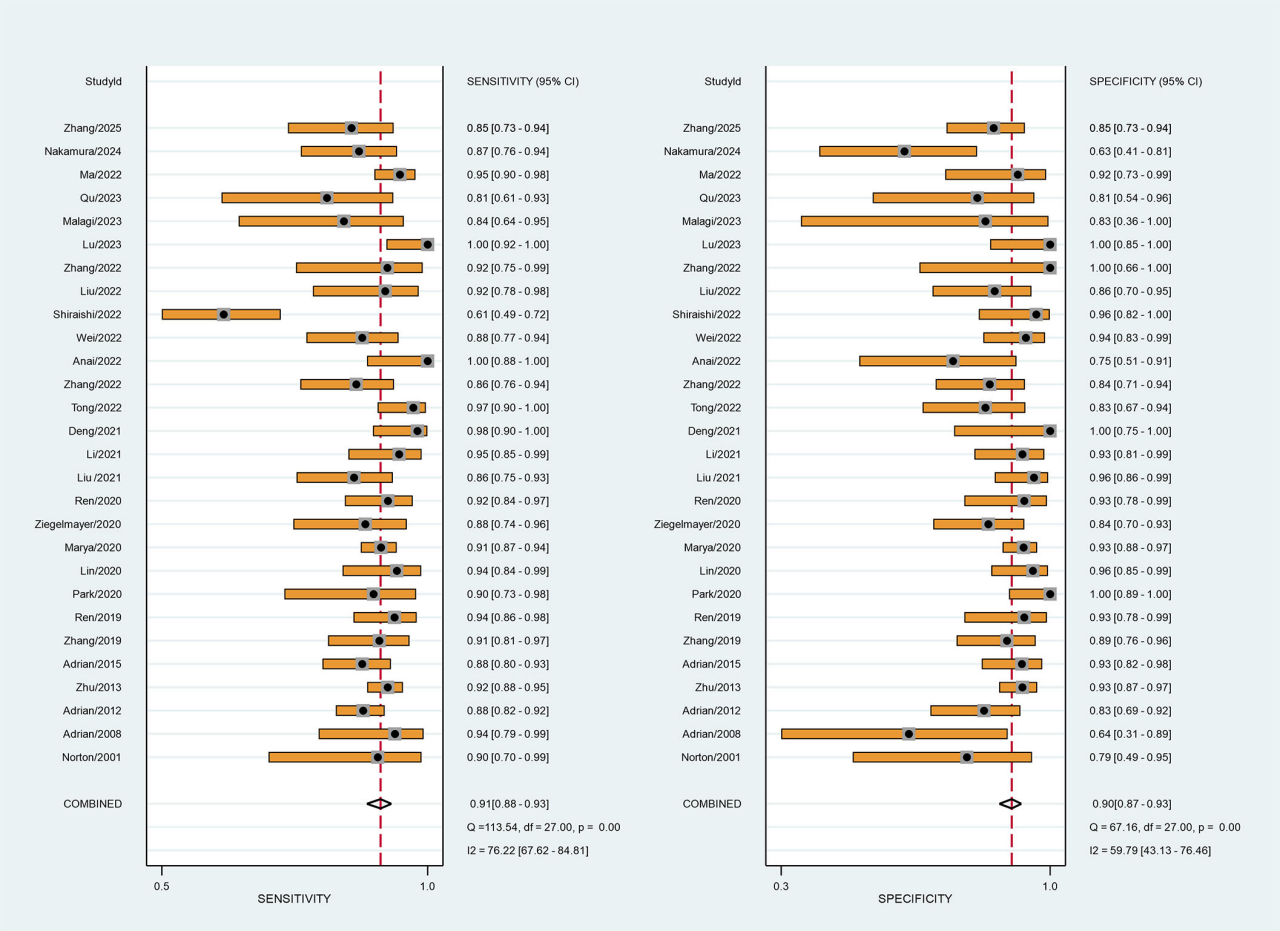
Forest plot of studies when selecting contingency tables reporting the Sen and Spe (28 studies with 28 best values).

The summary receiver operating characteristic (SROC) curves for the 28 included studies, comprising 76 contingency tables, are presented in **Figures 2**, **3**. The pooled sensitivity (SEN) and specificity (SPE) were 89% (95% CI: 87%–90%) and 88% (95% CI: 86%–90%), respectively, with an AUC of 0.94 (95% CI: 0.92–0.96) for all AI algorithms. When the highest accuracy contingency table was selected from these 28 studies ( **Table 3**), the pooled SEN and SPE were 91% (95% CI: 88%–93%) and 90% (95% CI: 87%–93%), respectively, with an AUC of 0.96 (95% CI: 0.94–0.97), as shown in **Figures 2**, **4**. However, substantial heterogeneity was observed among the included studies, with SEN having an *I*² = 77.14% and SPE having an *I*² = 75.61%.

**Table 3:** The best diagnostic performance of image-based AI algorithm for differentiating PC from pancreatitis.

Author and year	Number of patients				AUC	Sensitivity	Specificity	Algorithm classifier
	TP	FP	FN	TN				
Norton 2001	19	3	2	11	83.00%	89.00%	79.00%	ANNs
Adrian 2008	30	4	2	7	84.70%	93.80%	63.60%	ANNs
Adrian 2012	185	8	26	39	94.00%	87.59%	82.94%	ANNs
Zhu 2013	242	9	20	117	94.20%	92.52%	93.03%	SVM
Adrian 2015	98	4	14	51	–	87.50%	92.72%	ANNs
Zhang 2019	59	5	6	40	89.28%	89.24%	89.33%	SVM
Ren 2019	74	2	5	28	98.00%	94.00%	92.00%	NA
Park 2020	26	0	3	33	95.20%	89.70%	100.00%	RF
Lin 2020	48	2	3	43	94.80%	93.30%	96.10%	RF
Marya 2020	266	10	26	136	–	90.00%	93.00%	CNNs
Ziegelmayer 2020	37	7	5	37	90.00%	89.00%	83.00%	CNNs
Ren 2020	73	2	6	28	93.30%	92.20%	94.20%	RF
Liu 2021	55	2	9	46	96.68%	85.31%	96.04%	SVM-RFE
Li 2021	52	3	3	39	97.00%	95.24%	92.73%	LASSO
Deng 2021	50	0	1	13	99.70%	98.00%	100.00%	SVM
Tong 2022	71	6	2	30	97.30%	97.30%	83.30%	DLR
Zhang 2022	57	7	9	38	93.00%	86.00%	84.00%	SVM-RFE
Anai 2022	30	5	0	15	92.00%	100.00%	75.00%	SVM
Wei 2022	56	3	8	45	96.40%	87.50%	93.00%	MF
Shiraishi 2022	47	1	30	27	78.40%	60.70%	96.10%	SVM
Liu 2022	34	5	3	30	97.30%	92.20%	87.10%	LASSO
Ma 2022	143	2	8	22	94.70%	91.70%	98.00%	LASSO
Zhang 2022	24	0	2	9	94.00%	91.67%	100.00%	LASSO
Lu 2023	45	0	0	22	95.00%	100.00%	100.00%	RF
Malagi 2023	21	1	4	5	77.00%	83.00%	76.00%	ANNs
Qu 2023	21	3	5	13	81.00%	80.72%	81.25%	LASSO
Nakamura 2024	53	9	8	15	87.00%	63.00%	85.00%	CNNs
Zhang 2025	47	8	8	47	86.00%	86.00%	95.00%	LASSO

ANNs, artificial neural networks; SVM, support vector machine; NA, not available.

RF, random forest; CNNs, convolutional neural networks; SVM-RFE, support vector machine–recursive feature elimination.

LASSO, least absolute shrinkage and selection operator; DLR, deep learning radiomics, MF, multidomain fusion.

### 3.4 Publication bias

**Figure 5:**
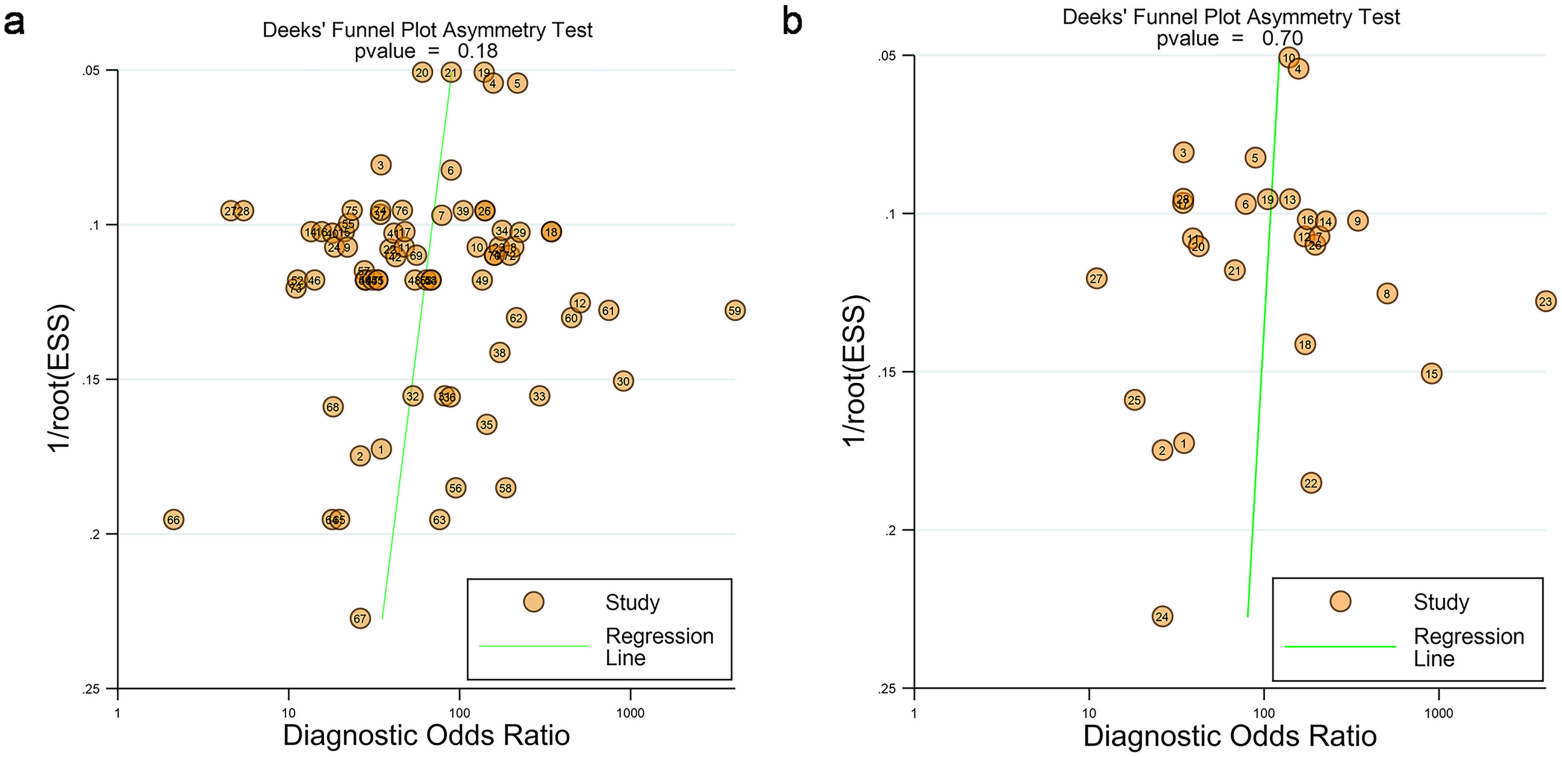
**(a)** Funnel plots of the 28 studies with 76 tables. **(b)** Funnel plots of studies when selecting contingency tables reporting the highest accuracy (28 studies with 28 best values).

Funnel plots indicated no significant publication bias among the 28 included studies with 76 contingency tables (*p* = 0.18) and when the highest accuracy contingency table was selected (*p* = 0.70), as illustrated in **Figures 5a, b**.

### 3.5 Subgroup and sensitive analyses

The detailed results of the subgroup analyses and exploration of potential sources of between-study heterogeneity are shown in **Table 4** and **Supplementary Figures S3–S20**. Considering the developmental stage and inherent differences of the algorithms, we categorized them into ML and DL groups and performed subgroup analyses. In the DL group, the pooled SEN and SPE were 89% (95% CI: 82%–93%) and 85% (95% CI: 76%–91%), respectively. In the ML group, the pooled SEN and SPE were 89% (95% CI: 86%–90%) and 88% (95% CI: 86%–90%), respectively (**Supplementary Figures S3a, b**, **S12**). For different imaging modality, nine US studies had a pooled SEN of 90% (95% CI: 88%–92%), a pooled SPE of 88% (95% CI: 83%–91%), and an AUC of 0.94 (95% CI: 0.92–0.96). Eleven CT studies had a pooled SEN of 89% (95% CI: 86%–92%), a pooled SPE of 90% (95% CI: 86%–93%), and an AUC of 0.96 (95% CI: 0.93–0.97). Four MRI studies had a pooled SEN of 88% (95% CI: 83%–92%), a pooled SPE of 86% (95% CI: 82%–89%), and an AUC of 0.89 (0.86–0.91). Four PET/CT studies had a pooled SEN of 85% (95% CI: 81%–87%), a pooled SPE of 86% (95% CI: 74%–93%), and an AUC of 0.87 (95% CI: 0.84–0.90) (**Supplementary Figures S4a–d**, **S13**). In eight studies using the same databases, AI had a pooled SEN of 88% (95% CI: 85%–91%), an SPE of 88% (95% CI: 85%–90%), and an AUC of 0.93 (95% CI: 0.91–0.95); however, clinician had a pooled SEN of 77% (95% CI: 66%–85%), an SPE of 80% (95% CI: 71%–87%), and an AUC of 0.85 (95% CI: 0.82–0.88) (**Supplementary Figures S5a, b**, **S14**). Twenty-one studies were conducted in Asia, and seven were conducted outside Asia. The pooled SEN and SPE were 88% (95% CI: 86%–90%) and 88% (95% CI: 85%–90%), respectively, with an AUC of 0.94 (95% CI: 0.92–0.96) in the Asia group. The pooled SEN and SPE were 90% (95% CI: 88%–91%) and 89% (95% CI: 85%–92%), respectively, with an AUC of 0.92 (95% CI: 0.90–0.94) (**Supplementary Figures S6a, b**, **S15**). Twenty-two studies had single-center data and six studies had multiple-center data. The pooled SEN and SPE were 88% (95% CI: 86%–90%) and 88% (95% CI: 85%–90%), respectively, with an AUC of 0.94 (95% CI: 0.92–0.96) in the single-center group. The pooled SEN and SPE were 91% (95% CI: 86%–94%) and 88% (95% CI: 84%–92%), respectively, with an AUC of 0.95 (95% CI: 0.92–0.96) (**Supplementary Figures S7a, b****S16**). Fourteen studies had sample sizes ≤100 and 14 studies had sample sizes >100. The pooled SEN and SPE were 87% (95% CI: 85%–90%) and 88% (95% CI: 84%–91%), respectively, and the AUC was 0.94 (95% CI: 0.91–0.95) for ≤100. For sample size >100, the pooled SEN and SPE were 90% (95% CI: 87%–92%) and 88% (95% CI: 85%–90%), respectively, for >100 with an AUC of 0.95 (95% CI: 0.92–0.96) (**Supplementary Figures S8a, b****S17**). Seven studies were published before 2020 and 21 studies were published after 2020. The pooled SEN was 90% (95% CI: 86%–93%) for studies published before 2020, and 90% (95% CI: 86%–93%) for those published after 2020. The SPE was 88% (95% CI: 86%–90%) and 88% (95% CI: 85%–90%), respectively. The AUC was 0.95 (95% CI: 0.93–0.97) and 0.94 (95% CI: 0.92–0.96), respectively (**Supplementary Figures S9a, b****S18**). Nine studies were low and 19 studies were high and unclear. The pooled SEN was 89% (95% CI: 85%–91%) for the high and unclear group, and 89% (95% CI: 84%–93%) for the low group. The SPE was 88% (95% CI: 86%–90%) and 88% (95% CI: 85%–90%), respectively. The AUC was 0.95 (95% CI: 0.92–0.96) and 0.94 (95% CI: 0.92–0.94), respectively (**Supplementary Figures S10a, b****S19**). Six studies used LASSO, six used SVM, and five used ANNs. The pooled SEN and SPE were 86% (95% CI: 83–89%) and 87% (95% CI: 84–90%), respectively, for the LASSO group (AUC 0.92, 95% CI: 0.90–0.94). For the SVM group, the pooled SEN and SPE were 95% (95% CI: 90–98%) and 87% (95% CI: 82–94%), respectively (AUC 0.97, 95% CI: 0.95–0.98). For the ANN group, the pooled SEN and SPE were 88% (95% CI: 84–91%) and 84% (95% CI: 73–91%), respectively (AUC 0.90, 95% CI: 0.87–0.93) (**Supplementary Figures S11a–c****S20**). Sensitive analysis results are shown in **Supplementary Table S5**.

**Table 4:** Subgroup analysis results.

Analysis	No. of trials	No. of tables	Sensitivity	I2 (%)	Specificity	I2 (%)	DOR	AUROC
Overall	28	76	0.89 [0.87, 0.90]	77.14	0.88 [0.86, 0.90]	75.61	58 [43, 76]	0.94 [0.92, 0.96]
Algorithm								
ML	25	69	0.89 [0.86, 0.90]	77.90	0.88 [0.86, 0.90]	76.69	59 [43, 79]	0.95 [0.92, 0.96]
DL	3	7	0.89 [0.82, 0.93]	75.35	0.85 [0.76, 0.91]	29.65	46 [22, 98]	0.94 [0.91, 0.95]
Imaging modality								
US	9	16	0.90 [0.88, 0.92]	54.18	0.88 [0.83, 0.91]	69.71	63 [42, 97]	0.94 [0.92, 0.96]
CT	11	33	0.89 [0.86, 0.92]	82.85	0.90 [0.86, 0.93]	73.43	77 [46, 128]	0.96 [0.93, 0.97]
MRI	4	18	0.88 [0.83, 0.92]	78.68	0.86 [0.82, 0.89]	15.83	45 [29, 70]	0.89 [0.86, 0.91]
PET	4	9	0.85 [0.81, 0.87]	29.43	0.86 [0.74, 0.93]	91.07	34 [14, 80]	0.87 [0.84, 0.90]
AI *vs*. clinician								
Clinician	8	14	0.77 [0.66, 0.85]	88.13	0.80 [0.71, 0.87]	75.78	13 [7, 22]	0.85 [0.82, 0.88]
AI	8	32	0.88 [0.85, 0.91]	80.44	0.88 [0.85, 0.90]	44.75	54 [37, 79]	0.93 [0.91, 0.95]
Geographical distribution								
Asia	21	67	0.88 [0.86, 0.90]	78.67	0.88 [0.85, 0.90]	77.22	57 [41, 78]	0.94 [0.92, 0.96]
Non-Asia	7	9	0.90 [0.88, 0.91]	0.00	0.89 [0.85, 0.92]	59.61	71 [46, 109]	0.92 [0.90, 0.94]
Center								
Single	22	60	0.88 [0.86, 0.90]	75.33	0.88 [0.85, 0.90]	78.37	52 [38, 73]	0.94 [0.92, 0.96]
Multiple	6	16	0.91 [0.86, 0.94]	83.77	0.88 [0.84, 0.92]	46.44	72 [45, 115]	0.95 [0.92, 0.96]
Sample size								
<100	14	26	0.87 [0.85, 0.90]	79.35	0.88 [0.84, 0.91]	84.97	52 [33, 81]	0.94 [0.91, 0.95]
>100	14	50	0.90 [0.87, 0.92]	76.48	0.88 [0.85, 0.90]	64.65	62 [44, 89]	0.95 [0.92, 0.96]
Year of publication								
Before 2020	7	11	0.90 [0.86, 0.93]	71.28	0.90 [0.86, 0.93]	52.20	75 [44, 129]	0.95 [0.93, 0.97]
After 2020	21	65	0.88 [0.86, 0.90]	77.80	0.88 [0.85, 0.90]	77.06	56 [40, 76]	0.94 [0.92, 0.96]
Risk of bias and concern of applicability of study								
Low	9	30	0.89 [0.85, 0.91]	80.03	0.89 [0.84, 0.93]	87.49	63 [34, 116]	0.95 [0.92, 0.96]
High + unclear	19	46	0.88 [0.86, 0.90]	75.83	0.88 [0.85, 0.90]	78.29	54 [38, 77]	0.94 [0.92, 0.96]
AI algorithm								
LASSO	6	25	0.86 [0.83, 0.89]	65.60	0.87 [0.84, 0.90]	24.27	42 [30, 58]	0.92 [0.90, 0.94]
SVM	6	11	0.95 [0.90, 0.98]	90.70	0.87 [0.82, 0.94]	75.10	165 [97, 283]	0.97 [0.95, 0.98]
ANNS	5	5	0.88 [0.84, 0.91]	0.00	0.84 [0.73, 0.91]	43.79	39 [20, 77]	0.90 [0.87, 0.93]

ML, machine learning; DL, deep learning.

## 4 Discussion

Focal inflammatory pancreatic lesions (FIPLs) are localized inflammatory masses arising from acute or chronic pancreatitis and often mimic PC on imaging, leading to diagnostic uncertainty. Accurately distinguishing PC from FIPLs remains clinically important because the two conditions require fundamentally different management strategies. This study aims to comprehensively assess the use of AI algorithms for differentiating PC from pancreatitis. Although the clinical symptoms of PC and pancreatitis patients are similar, their treatment needs and survival rates differ. Therefore, it is necessary for clinicians to differentiate between PC and pancreatitis. A large number of studies have been conducted in the last decade to differentiate PC from pancreatitis by extracting and analyzing imaging features and conduct AI algorithms due to the rapid growth of AI technology. We conducted this study to provide the highest level of evidence to assess the feasibility of AI to promote precision medicine in PC treatment. To our knowledge, this is the first systematic review and meta-analysis of the diagnostic performance of AI algorithms in this field. After a careful selection of research on relevant topics, we found that AI algorithms excelled in differentiating PC from pancreatitis using medical radiography imaging with a pooled SEN of 89% (95% CI: 87%–90%), a pooled SPE of 88% (95% CI: 86%–90%), and an AUC of 0.94 (95% CI: 0.92–0.96) in 28 included studies with 76 contingency tables, which manifested better performance than independent detection with a pooled SEN of 91% (95% CI: 88%–93%), a pooled SPE of 90% (95% CI: 87%–93%), and an AUC of 0.96 (95% CI: 0.94–0.97) in 28 included studies with 28 best value tables.

### 4.1 Key findings and clinical implications

The high diagnostic accuracy of AI algorithms in this meta-analysis highlights their potential to complement or even surpass conventional imaging methods. The overlapping clinical and imaging features of PC and pancreatitis often lead to diagnostic uncertainty, which can result in delayed treatment for PC or unnecessary surgery for pancreatitis (50, 51). AI’s ability to extract and analyze high-throughput quantitative features from imaging data offers a promising solution to this problem (52). Notably, the pooled SEN and SPE of AI algorithms exceeded the performance thresholds typically observed in conventional imaging, suggesting that AI could serve as a valuable decision-support tool in clinical practice (53).

Subgroup analyses revealed that both ML and DL algorithms demonstrated comparable diagnostic efficacy, with ML achieving an SEN of 89% and an SPE of 88%, and DL achieving an SEN of 90% and an SPE of 88%. This suggests that the choice of algorithmic architecture may depend on specific clinical contexts and available computational resources. When stratified by imaging modality, AI models demonstrated generally high diagnostic performance across all four modalities. CT-based models achieved the highest pooled accuracy (AUC 0.95), indicating excellent discriminative ability. Ultrasound-based AI also showed strong performance (AUC 0.92), followed by MRI-based approaches (AUC 0.89). PET/CT-based AI yielded a somewhat lower accuracy (AUC 0.87) compared with the other modalities, although its performance remained clinically meaningful. These findings indicate that while AI can provide valuable diagnostic support across different imaging modalities, performance levels are not uniform. The inclusion of heterogeneous imaging modalities likely contributed to the overall inconsistency observed in the meta-analysis. Future research with larger, modality-specific datasets will be needed to determine the optimal imaging context for AI application in distinguishing PC from pancreatitis.

When analyzed separately, AI demonstrated generally high diagnostic performance across all four imaging modalities, although important differences were observed. CT-based AI models achieved the highest pooled accuracy with an AUC of 0.96, an SEN of 89% (95% CI: 86%–92%), and an SPE of 90% (95% CI: 86%–93%), and showed relatively higher heterogeneity (*I*² = 82.85% in SEN and *I*² = 73.43% in SPE). This strong performance may be attributed to the widespread availability of CT, larger training datasets, and more standardized acquisition protocols in the included studies.

Ultrasound-based AI also performed well, with an AUC of 0.94, an SEN of 90% (95% CI: 88%–92%), and an SPE of 88% (95% CI: 83%–91%), although heterogeneity was moderate (*I*² = 54.18% in SEN and *I*² = 69.71% in SPE). This variability likely reflects operator dependence and inconsistent image quality across centers. The relatively strong performance despite these limitations suggests that AI may help mitigate inter-operator variability by standardizing interpretation, though most studies were single-center with limited sample sizes.

MRI-based models reached a pooled AUC of 0.89, an SEN of 88% (95% CI: 83%–92%), and an SPE of 86% (95% CI: 82%–89%), but showed high heterogeneity (*I*² = 78.68% in SEN and *I*² = 15.83% in SPE). While MRI offers superior soft-tissue contrast, the diversity of acquisition sequences (T1WI, T2WI, and DWI) and lack of harmonization across institutions likely contributed to the variability. Standardized MRI protocols will be critical for improving reproducibility in future studies.

PET/CT-based AI demonstrated the lowest pooled accuracy with an AUC of 0.87, an SEN of 85% (95% CI: 81%–87%), and an SPE of 86% (95% CI: 74%–93%), accompanied by substantial heterogeneity (*I*² = 29.43% in SEN and *I*² = 91.07% in SPE). Small study numbers, differences in tracer selection, and heterogeneous reconstruction protocols may explain these results.

Collectively, these findings indicate that although AI shows diagnostic value across modalities, the magnitude of benefit is not uniform. The overall high heterogeneity (*I*² > 75%) in our meta-analysis can be explained, at least in part, by modality-specific differences. From a clinical perspective, CT- and ultrasound-based AI appear most promising for near-term application due to higher accuracy and accessibility, whereas MRI- and PET/CT-based approaches will require larger, multicenter datasets and standardized acquisition protocols to reduce heterogeneity and establish reliable clinical utility.

In our subgroup analysis by algorithm type, SVM-based models achieved the highest pooled diagnostic accuracy (AUC 0.97), followed by LASSO and ANN frameworks. Although these differences were not statistically significant, the trend suggests that kernel-based classifiers such as SVM may be particularly effective for radiomics-derived imaging features, whereas ANN performance may be limited by small sample sizes and non-standardized input data.

### 4.2 Comparison with human clinicians

A notable finding from this meta-analysis is the consistently higher pooled diagnostic performance of AI compared with clinicians in studies that directly evaluated both approaches using the same patient datasets. Across eight studies, AI achieved a pooled sensitivity of 88% and a pooled specificity of 88% (AUC 0.93), whereas clinicians achieved a pooled sensitivity of 77% and a pooled specificity of 80% (AUC 0.85). These results suggest that AI systems may provide comparable or numerically higher diagnostic accuracy than clinicians in differentiating PC from pancreatitis. However, this difference should be interpreted cautiously, as the included studies were heterogeneous in imaging modality, algorithm type, and clinician experience, and lacked formal paired statistical testing. Rather than replacing clinicians, AI may help reduce inter-reader variability and enhance diagnostic consistency, especially in settings with limited subspecialty expertise. However, it is important to note that AI systems are not intended to replace clinicians but rather to augment their diagnostic capabilities, particularly in complex cases where imaging features are ambiguous. However, this advantage was not consistent across all modalities and study settings, and several reports indicated comparable performance between AI and radiologists. These findings suggest that AI may complement clinicians by providing reproducible, quantitative assessments, but it cannot yet be regarded as superior or a replacement. For clinical practice, AI could be particularly useful in cases where imaging features are subtle or inter-observer variability is high, offering an additional layer of decision support. Still, the variability in algorithm design, training datasets, and external validation highlights the need for caution. Future multicenter prospective trials are required to determine whether AI can achieve robust and generalizable improvements in real-world workflows, and to clarify the conditions under which AI provides incremental benefit over expert interpretation (54).

### 4.3 Impact of study quality on robustness

The sensitivity analysis demonstrated that the pooled diagnostic accuracy of AI was largely unaffected by the exclusion of studies with high risk of bias. The recalculated estimates for sensitivity, specificity, DOR, and AUROC were nearly identical to those of the primary analysis, reinforcing the stability and reliability of the overall findings as shown in **Supplementary Table S5**. Interestingly, although slight numerical differences were observed—particularly a marginal increase in DOR and AUROC after excluding high-bias studies—these changes were not substantial. This trend may indicate that higher-quality studies tend to yield more consistent and slightly stronger diagnostic performance, possibly due to standardized patient selection and imaging protocols.

Nevertheless, caution is warranted, as such differences could also arise from variations in sample size, imaging modality, or algorithm type rather than methodological quality alone. Overall, the consistency of the results across sensitive analyses supports the robustness of the pooled conclusions and underscores the importance of maintaining methodological rigor in future AI-based diagnostic research.

### 4.4 Sources of heterogeneity and limitation

Despite the promising results, significant heterogeneity was observed among the included studies (*I*² > 75%). Subgroup analyses identified several factors contributing to this heterogeneity, including algorithmic architecture, imaging modality, geographic origin, and publication year. For instance, studies conducted in Asia demonstrated slightly higher diagnostic accuracy (AUC = 0.95) compared to those outside Asia (AUC = 0.92). Similarly, single-center studies showed marginally better performance than multi-center studies, possibly due to standardized imaging protocols and reduced variability in data collection (54). These findings suggest that modality-specific factors, such as image resolution, acquisition protocols, and dataset availability, may partly explain the inconsistency observed in the overall pooled results. Nevertheless, the number of studies per modality remains limited, which restricts the ability to draw firm conclusions. Future studies focusing on single-modality AI applications, ideally with larger and more standardized datasets, will be essential to clarify the role of imaging modality in diagnostic performance.

There are several limitations in our meta-analysis. Firstly, almost all of the included studies were retrospective, which introduced potential bias in patient selection. Secondly, we manually calculated the data necessary to construct the confusion matrix in some studies due to the absence of full test in performance metrics. Additionally, some studies were abandoned as we failed to extract or calculate numbers of TN, TP, FP, and FN. We should be cautious in interpreting pooled estimates from quantitative analyses. Thirdly, despite the implementation of subgroup analyses and sensitivity tests, the sources of heterogeneity still waited for further detection. Similar high heterogeneity was also observed in recent systematic reviews evaluating the quality of radiomics models in other fields.

### 4.5 Future directions and conclusion

In summary, AI algorithms demonstrate robust diagnostic performance in differentiating PC from pancreatitis with CT- and ultrasound-based approaches currently appearing most promising for clinical translation. Importantly, AI should be viewed as an adjunct to, rather than a replacement for, radiologists, providing quantitative, reproducible insights that can support clinical decision-making. Future research should focus on prospective multicenter studies with standardized imaging protocols, external validation, and explainable AI frameworks to facilitate integration into radiology workflows. Combining AI with other data modalities, such as genomic or histopathologic information, may further enhance diagnostic accuracy and pave the way for precision medicine in pancreatic diseases.

## Data Availability

The original contributions presented in the study are included in the article/**Supplementary Material**. Further inquiries can be directed to the corresponding authors.
