# Supplementary material for: Image-based artificial intelligence for preoperative differentiation of pancreatic cancer from pancreatitis: a systematic review and meta-analysis
Source: Front Oncol. 2026 Jan 12;15:1660271. doi: 10.3389/fonc.2025.1660271 (PMC12832336; doi:10.3389/fonc.2025.1660271)
Supplement: Supplementary file 1 [file DataSheet1.docx]

*Supplementary Materials*

**Image-Based Artificial Intelligence for Preoperative Differentiation of Pancreatic Cancer from Pancreatitis: A Systematic Review and Meta-Analysis**

**Contents**

**Table S1:** Literature searching strategies in PubMed, Embase and Cochrane Library database.

**Table S2:** Detail characteristics of the 28 included studies with 76 contingency tables.

**Table S3:** Detail characteristics of the 28 included studies with 28 best diagnostic performance tables.

**Table S4:** The pooled sensitivity, specificity and AUC of different groups.

**Table S5:** The sensitivity analysis results of different groups.

**Fig S1:** Supplementary Figure 1. QUADAS-2 summary plot.

**Fig S2:** Risk of bias and concern of applicability for each item in included.

**Fig S3:** SROC curve of studies using different algorithms (DL or ML).

**Fig S4:** SROC curve of different imaging modalities (US, CT, MRI, or PET).

**Fig S5:** SROC curve of AI vs Clinician in same dataset (AI vs Clinician).

**Fig S6:** SROC curve of different geographical distribution (Asia or non Asia).

**Fig S7:** SROC curve of different centers (single or multiple ).

**Fig S8:** SROC curve of studies with different sample size(≤ 100 or > 100).

**Fig S9:** SROC curve of different publication year (before or after 2020).

**Fig S10:** SROC curve of different risk of bias levels (High/Unclear or Low).

**Fig S11:** SROC curve of different AI algorithm.

**Fig S12:** Forest plot of different studies using different algorithms (DL or ML).

**Fig S13:** Forest plot of different imaging modalities (US, CT, MRI, or PET).

**Fig S14:** Forest plot of AI vs Clinician in same dataset (AI vs Clinician).

**Fig S15:** Forest plot of different geographical distribution (Asia or non Asia).

**Fig S16:** Forest plot of different centers (Single or multiple).

**Fig S17:** Forest plot of different sample sizes (≤ 100 or > 100).

**Fig S18:** Forest plot of different publication year (before or after 2020).

**Fig S19:** Forest plot of different risk of bias levels (High/Unclear or Low).

**Fig S20:** Forest plot of different AI algorithm.

**Table S1:** Detail literature searching strategies in PubMed, Embase and Cochrane Library database.

| **Databases** |  | **Retrieval strategy** | **Items found** |
| --- | --- | --- | --- |
| **PubMed** | #1 | "Pancreatitis"[MeSH Terms] OR "Pancreatitis"[Title/Abstract] OR "Pancreatitides"[Title/Abstract] OR "autoimmune pancreatitis"[Title/Abstract] | 83179 |
|  | #2 | "pancreas cancer"[MeSH Terms] OR "pancreas cancer"[Title/Abstract] OR "pancreas carcinoma"[Title/Abstract] OR "pancreas cancers"[Title/Abstract] OR "pancreas neoplasm"[Title/Abstract] OR "pancreas malignan"[Title/Abstract]OR "pancreatic cancer"[Title/Abstract] OR "pancreatic carcinoma"[Title/Abstract] OR "pancreatic cancers"[Title/Abstract] OR "pancreatic neoplasm"[Title/Abstract] OR "pancreatic malignan"[Title/Abstract] OR "PDAC"[Title/Abstract] OR "pancreatic ductal adenocarcinoma"[Title/Abstract] | 77303 |
|  | #3 | "Artificial Intelligence"[MeSH Terms] OR "artificial intelligence"[Title/Abstract] OR "computational intelligence"[Title/Abstract] OR "machine intelligence"[Title/Abstract] OR "AI"[Title/Abstract] OR "computer vision systems"[Title/Abstract] OR "computer vision system"[Title/Abstract] OR "knowledge acquisition"[Title/Abstract] OR "knowledge representation"[Title/Abstract] OR "knowledge representations"[Title/Abstract] | 351324 |
|  | #4 | Machine Learning[MeSH Terms] OR "machine learning"[Title/Abstract] OR "transfer learning"[Title/Abstract] | 214222 |
|  | #5 | "Deep Learning"[MeSH Terms] OR "deep learning"[Title/Abstract] OR "hierarchical learning"[Title/Abstract] | 98802 |
|  | #6 | Supervised Machine Learning[MeSH Terms] OR "supervised machine learning"[Title/Abstract] OR "semi supervised learning"[Title/Abstract] OR "inductive machine learning"[Title/Abstract] OR "active machine learning"[Title/Abstract] | 19564 |
|  | #7 | #3 OR #4 OR #5 OR #6 | 467989 |
|  | #8 | Diagnosis[MeSH Terms] OR "diagnosis"[Title/Abstract]OR "sensitivity"[Title/Abstract] OR "specificity"[Title/Abstract] OR "accuracy"[Title/Abstract] OR "differential"[Title/Abstract] OR "differential"[Title/Abstract] OR "differentiation"[Title/Abstract]OR "diagnostic performance"[Title/Abstract] | 12908050 |
|  | #9 | ("1965/7/10"[Date - Publication] : "2025/6/30"[Date - Publication]) | 36603854 |
|  | #10 | #1 AND #2 AND #3 AND #7 AND #8 AND #9 | 92 |
| **Embase** | #1 | artificial intelligence'/exp OR 'artificial intelligence':ab,ti OR 'computational intelligence':ab,ti OR 'machine intelligence':ab,ti OR 'ai':ab,ti OR 'computer vision systems':ab,ti OR 'computer vision system':ab,ti OR 'knowledge acquisition':ab,ti OR 'knowledge representation':ab,ti OR 'knowledge representations':ab,ti | 215646 |
|  | #2 | machine learning'/exp OR 'machine learning':ab,ti OR 'transfer learning':ab,ti | 596521 |
|  | #3 | deep learning'/exp OR 'deep learning':ab,ti OR 'hierarchical learning':ab,ti | 78621 |
|  | #4 | supervised machine learning'/exp OR 'supervised machine learning':ab,ti OR 'semi supervised learning':ab,ti OR 'inductive machine learning':ab,ti OR 'active machine learning':ab,ti | 7658 |
|  | #5 | #1 OR #2 OR #3 OR #4 | 671631 |
|  | #6 | pancreatitis'/exp OR 'pancreatitis':ab,ti OR 'pancreatitides':ab,ti OR 'autoimmune pancreatitis':ab,ti | 183165 |
|  | #7 | pancreas cancer'/exp OR 'pancreas cancer':ab,ti OR 'pancreas carcinoma':ab,ti OR 'pancreas cancers':ab,ti OR 'pancreas neoplasm':ab,ti OR 'pancreas malignan':ab,ti OR 'pancreatic cancer':ab,ti OR 'pancreatic carcinoma':ab,ti OR 'pancreatic cancers':ab,ti OR 'pancreatic neoplasm':ab,ti OR 'pancreatic malignan':ab,ti OR 'pdac':ab,ti OR 'pancreatic ductal adenocarcinoma':ab,ti | 186156 |
|  | #8 | diagnosis'/exp OR 'diagnosis':ab,ti OR 'sensitivity':ab,ti OR 'specificity':ab,ti OR 'accuracy':ab,ti OR 'differential':ab,ti OR 'differentiation':ab,ti OR 'diagnostic performance':ab,ti | 13581136 |
|  | #9 | #5 AND #6 AND #7 AND #8 | 313 |
| **Cochrane Library** | #1 | MeSH descriptor: [Artificial Intelligence] explode all trees | 3056 |
|  | #2 | (Artificial Intelligence):ti,ab,kw OR (Computational Intelligence):ti,ab,kw OR (Machine Intelligence):ti,ab,kw OR (AI):ti,ab,kw OR (Computer Vision Systems):ti,ab,kw OR (Computer Vision System):ti,ab,kw OR (Knowledge Acquisition):ti,ab,kw | 0 |
|  | #3 | #1 OR #2 | 3115 |
|  | #4 | (MeSH descriptor: [Machine Learning] explode all trees | 989 |
|  | #5 | (Machine Learning):ti,ab,kw OR (Transfer Learning):ti,ab,kw | 4,356 |
|  | #6 | #4 OR #5 | 1 |
|  | #7 | MeSH descriptor: [Deep Learning] explode all trees | 291 |
|  | #8 | (Deep Learning):ti,ab,kw OR (Hierarchical Learning):ti,ab,kw | 1715 |
|  | #9 | #7 OR #8 | 1764 |
|  | #10 | #3 OR #6 OR #9 | 5123 |
|  | #11 | MeSH descriptor: [Pancreatitis] explode all trees | 1958 |
|  | #12 | (Pancreatitis):ti,ab,kw OR (Pancreatitides):ti,ab,kw OR (Autoimmune Pancreatitis):ti,ab,kw | 5684 |
|  | #13 | #11 OR #12 | 5561 |
|  | #14 | MeSH descriptor: [Pancreas Cancer] explode all trees | 2569 |
|  | #15 | (Pancreas Cancer):ti,ab,kw OR (Pancreas Carcinoma):ti,ab,kw OR (Pancreas Cancers):ti,ab,kw OR (Pancreas Neoplasm):ti,ab,kw OR (Pancreas Malignan):ti,ab,kw OR (Pancreatic Cancer):ti,ab,kw OR (Pancreatic Carcinoma):ti,ab,kw OR (Pancreatic Cancers):ti,ab,kw OR (Pancreatic Neoplasm):ti,ab,kw OR (Pancreatic Malignan):ti,ab,kw OR (PDAC):ti,ab,kw OR (Pancreatic Ductal Adenocarcinoma):ti,ab,kw | 7156 |
|  | #16 | #14 OR #15 | 7765 |
|  | #17 | MeSH descriptor: [Diagnosis] explode all trees | 1789 |
|  | #18 | (Diagnosis):ti,ab,kw OR (Sensitivity):ti,ab,kw OR (Specificity):ti,ab,kw OR (Accuracy):ti,ab,kw OR (Differential):ti,ab,kw OR (Differentiation):ti,ab,kw OR (Diagnostic performance):ti,ab,kw | 294635 |
|  | #19 | #14 OR #15 | 7538 |
|  | #20 | #10 AND #13 AND #16 AND #19 | 3 |

**Table S2:** Detail characteristics of the 28 included studies with 76 contingency tables.

| **Author** | **Year** | **TP** | **FP** | **FN** | **TN** | **Sensitivity** | **Specificity** | **Imaging modality** | **Algorithm architecture** | **Feature** | **Radiomics software** | **Seg Software** | **Scanner** | **Parameter** |
| --- | --- | --- | --- | --- | --- | --- | --- | --- | --- | --- | --- | --- | --- | --- |
| Norton | 2001 | 19 | 3 | 2 | 11 | 89.00% | 79.00% | EUS | ANNs | NR | NR | NR | radial US system (EUM20, Olympus America, Inc., Melville, NY) | NR |
| Adrian | 2008 | 30 | 4 | 2 | 7 | 93.80% | 63.60% | EUS | ANNs | NR | NR | NR | US system with an embedded Sonoelastography module (Hitachi 8500; Hitachi Medical Systems Europe Holding AG, Zug, Switzerland), used in conjunction with a Pentax linear endoscope (EG 3830UT or EG 3870 UTK; Pentax, Hamburg, Germany). | NR |
| Adrian | 2012 | 185 | 8 | 26 | 39 | 87.59% | 82.94% | EUS | ANNs | NR | NR | NR | NR | NR |
| Zhu | 2013 | 242 | 9 | 20 | 117 | 92.52% | 93.03% | EUS-Half-and-half method | SVM | 105 | NR | NR | EndoEcho UM 2000 ultrasonic endoscope (Olympus Corporation, Tokyo, Japan) with a probe frequency of 7.5 MHz. | NR |
|  |  | 240 | 6 | 22 | 120 | 91.55% | 95.07% | EUS-Leave-one-out method | SVM | 105 | NR | NR |  |  |
| Adrian | 2015 | 98 | 4 | 14 | 51 | 87.50% | 92.72% | EUS | ANNs | NR | NR | NR | The protocol of EUS with EUSFNA included linear EUS instruments (both Pentax UTK 3870 [Pentax, Japan] and Olympus GF-UCT 180 [Olympus, Japan], coupled with the corresponding Hitachi Preirus [Hitachi, Japan] and Aloka alpha 5 US systems [Hitachi, Japan]) | NR |
| Zhang | 2019 | 59 | 5 | 6 | 40 | 89.24% | 89.33% | PET/CT | SVM | 418 | NR | NR | NR | NR |
| Ren | 2019 | 74 | 2 | 5 | 28 | 94.00% | 92.00% | CT-Model 1 | NA | 396 | NR | NR | Multi-detector CT systems, including Philips Brilliance 64  (Philips Healthcare, DA Best, the Netherlands) and Optima 670 (GE Healthcare, Tokyo, Japan) | CT scanning parameters were as follows: tube voltage, 120 kVp; current, 200–400 mAs; pitch, 1.375; rotation speed, 0.75 s; slice thickness, 3.0 mm; slice interval, 3.0 mm; and a reconstruction interval of 1.25 mm |
|  |  | 61 | 4 | 18 | 26 | 77.00% | 86.00% | CT-Model 2 | NA | 396 | NR | NR |  |  |
|  |  | 76 | 5 | 3 | 25 | 96.00% | 83.00% | CT-Model 3 | NA | 396 | NR | NR |  |  |
|  |  | 61 | 2 | 18 | 28 | 77.00% | 94.00% | CT-Model 1+2+3 | NA | 396 | NR | NR |  |  |
| Park | 2020 | 26 | 0 | 3 | 33 | 89.70% | 100.00% | CT | RF | 431 | NR | Velocity AITM (Varian Medical Systems), | Sixty-five patients with AIP were scanned with a dual-source MDCT scanner (Somatom Definition®, Definition Flash®, or Force®, Siemens Healthineers) and the remaining 24 patients were scanned on a 16- or 64-slice MDCT scanner (Somatom Sensation® 16 or 64, Siemens Healthineers). | 100–120 mL of iohexol (OmnipaqueTM, General Electric Healthcare). injection rate of 4–5 mL/s 120 kVp, 300 mAs.The collimation was 128 × 0.6 mm or 192 × 0.6 mm for the dual source scanner, 16 × 0.75 mm for the 16-slice scanner, and 64 × 0.6 mm for the 64-slice scanner |
| Lin | 2020 | 48 | 2 | 3 | 43 | 93.30% | 96.10% | CT-Radiomics: hybrid | RF | 1160 | NR | MATLAB 2016b (MathWorks, Natick, Massachusetts). | helical multidetector (16, 64, 128, and 256 slices) CT scanners | After an unenhanced scanning (120 kVp, 100–500 mAs), dual-phase (arterial and venous) upper abdomen imaging was acquired using an intravenous bolus injection of iodinated contrast (1 mL/kg) with a 3- to 4-mL/s injection rate |
|  |  | 29 | 4 | 22 | 41 | 57.78% | 90.20% | CT-Radiologist 1 | RF | 1160 | NR | NR |  |  |
|  |  | 37 | 5 | 14 | 40 | 73.33% | 88.24% | CT-Radiologist 2 | RF | 1160 | NR | NR |  |  |
|  |  | 36 | 6 | 15 | 39 | 71.11% | 86.27% | CT-Radiomics: noncontrast | RF | 1160 | NR | NR |  |  |
|  |  | 42 | 4 | 9 | 41 | 82.22% | 90.20% | CT-Radiomics: arterial phase | RF | 1160 | NR | NR |  |  |
|  |  | 48 | 2 | 3 | 43 | 93.33% | 96.08% | CT-Radiomics: venous phase | RF | 1160 | NR | NR |  |  |
| Marya | 2020 | 266 | 10 | 26 | 136 | 90.00% | 93.00% | EUS-Alll stills | CNNs | NR | NR | NR | NR | NR |
|  |  | 263 | 19 | 29 | 127 | 90.00% | 87.00% | EUS | CNNs | NR | NR | NR |  |  |
|  |  | 266 | 15 | 26 | 131 | 91.00% | 90.00% | EUS-Videos only | CNNs | NR | NR | NR |  |  |
| Ziegelmayer | 2020 | 37 | 7 | 5 | 37 | 89.00% | 83.00% | CT | CNNs | 1411 | NR | NR | NR | NR |
| Ren | 2020 | 73 | 2 | 6 | 28 | 92.20% | 94.20% | CT -RF | RF | 396 | NR | NR | Philips Brilliance 64 (Philips Healthcare, DA Best, the Neth  erlands) and Optima 670 (GE Healthcare, Tokyo, Japan) devices | The CT acquisition parameters were as following: slice thickness of 3 mm, slice interval of 3 mm, tube voltage of 120 kV, tuber current of 200–400 mA, gantry rotation  speed of 0.75 s, pitch of 1.375, and a reconstruction interval of 1.25 mm. |
|  |  | 65 | 6 | 14 | 24 | 82.60% | 80.80% | CT -LGOCV | LGOCV | 396 | NR | NR |  |  |
| Liu | 2021 | 55 | 2 | 9 | 46 | 85.31% | 96.04% | PET/CT (DTM model) | SVM-RFE | 502 | NR | NR | PET/CT scanner (Siemens  Biograph64). | fasting blood glucose concentration of < 11.1 mmol/L. 2-[18F]FDG (3.70–5.55 MBq/kg) was injected intravenously through the cubital vein and PET/CT imaging was performed after the patients laid quietly in the dark for 50–60 min. |
|  |  | 55 | 2 | 9 | 46 | 85.31% | 96.25% | PET/CT (Fusion model) | SVM-RFE | 502 | NR | NR |  |  |
|  |  | 50 | 21 | 14 | 27 | 77.50% | 55.29% | PET/CT (CD model) | SVM-RFE | 502 | NR | NR |  |  |
|  |  | 56 | 27 | 8 | 21 | 87.50% | 44.79% | PET/CT (doctors model) | SVM-RFE | 502 | NR | NR |  |  |
| Li | 2021 | 52 | 3 | 3 | 39 | 95.24% | 92.73% | CT | LASSO | 1409 | NR | NR | Multiphasic CT was performed by following a pancreas-specific protocol and using 256- and 320-slice multidetector row CT scanners (256: Brilliance-16P, Philips Healthcare, Cleveland, American; 320: Aquilion ONE, Canon Medical Systems, Tokyo, Japan) | The CT scan parameters were as follows: 120 kV, 150 effective mAs, beam collimation of 160 £ 0.5 mm, a matrix of 350 £ 350, and a gantry rotation time of 0.5 s. |
| Deng | 2021 | 50 | 0 | 1 | 13 | 98.00% | 100.00% | MRI (P model) | SVM | 1409 | NR | NR | 3.0-T MR examination (MR 750, GE Medical Systems, Waukesha, WI, USA, and Achieva, Philips, the Netherlands). | T1WI-TR:4, TE 2, Selection thickness 4; DCE-MRI-TR:4, TE 2, Selection thickness 4 |
|  |  | 49 | 3 | 2 | 10 | 96.10% | 76.90% | MRI (T1WI model) | SVM | 1409 | NR | NR |  |  |
|  |  | 48 | 3 | 3 | 10 | 94.10% | 76.90% | MRI (T2WI model) | SVM | 1409 | NR | NR |  |  |
|  |  | 49 | 1 | 2 | 12 | 96.10% | 92.30% | MRI (A model) | SVM | 1409 | NR | NR |  |  |
| Tong | 2022 | 71 | 6 | 2 | 30 | 97.30% | 83.30% | EUS -internal validation | DLR | NR | labelme | NR | Four different US devices (MyLab 90, ESAOTE, Italy; Aloka, HITACHI, Japan; LIGIQ E20, GE, USA; Resona  7, Mindray, China) equipped with an abdominal probe | 2.4 mL of the contrast agent (SonoVue®; Bracco, Milan, Italy) was injected, followed by a 5-mL saline flush. The timer  was started simultaneously when the contrast agent was being injected. Subsequently, the probe was kept in a stable state for 120 s |
|  |  | 34 | 0 | 5 | 11 | 87.20% | 100.00% | EUS-external validation cohort 1 | DLR | NR | labelme | NR |  |  |
|  |  | 32 | 4 | 1 | 11 | 97.40% | 70.00% | EUS-external validation cohort 2 | DLR | NR | labelme | NR |  |  |
| Zhang | 2022 | 57 | 7 | 9 | 38 | 86.00% | 84.00% | PET/CT | SVM-RFE | 251 | NR | NR | Siemens Biograph64 PET/CT (52-ring LSO crystal and 64-slice spiral CT) | Subjects were instructed to fast for at least 6 hours, and 3.70-5.55 MBq/kg of 18F-FDG  was intravenously injected when blood glucose (BG) < 11.1 mmol/L.  whole-body CT scans were performed using an electric current of 170 mA at a voltage of 120 kV, with a scan time of 18.67-21.93 s. |
| Anai | 2022 | 30 | 5 | 0 | 15 | 100.00% | 75.00% | CT | SVM | 62 | NR | NR | 16-slice MDCT scanner (Aquilion®, Toshiba Medical Systems, Tokyo, Japan), a 32-slice MDCT scanner (Aquilion®, Toshiba Medical Systems, Tokyo, Japan), a 64-slice MDCT scanner (Aquilion®, Toshiba Medical Systems, Tokyo, Japan), and a 320-slice MDCT scanner (Aquilion ONE®, Toshiba Medical Systems, Tokyo, Japan) | CT data of the 16-slice MDCT scanner were acquired using the following parameters: tube voltage, 120 kV; tube current, 200 mA without automatic exposure control;  CT data of the 32-slice MDCT scanner were acquired using the following parameters: tube  voltage, 120 kV; tube current, 270 mA without automatic  exposure control; |
| Wei | 2022 | 56 | 3 | 8 | 45 | 87.50% | 93.00% | PET/CT-MF | MF | NR | NR | NR | Siemens Biograph64 PET/CT scanner. | Before PET/CT scanning, patients should fast for at least 6 h, and 18F-FDG (3.70–5.55 MBq/kg) was intravenously injected when blood glucose<11.1 mmol/L. 30 mA at a voltage of 120 kV |
|  |  | 59 | 5 | 19 | 29 | 75.20% | 85.50% | PET/CT-RAD | RAD | NR | NR | NR |  |  |
|  |  | 66 | 4 | 12 | 30 | 84.40% | 87.90% | PET/CT-DL | DL | NR | NR | NR |  |  |
| Shiraishi | 2022 | 47 | 1 | 30 | 27 | 60.70% | 96.10% | MRI | SVM | NR | NR | NR | 1.5-Tesla MR unit (Magnetom Avanto or Symphony, Siemens Healthcare, Erlangen, Germany) with a 6-channel body matrix coil. | NR |
| Liu | 2022 | 34 | 5 | 3 | 30 | 92.20% | 87.10% | MRI (Mix model) | LASSO | 960 | NR | NR | 3.0-T MRI (MAGNETOM Skyra, Siemens Healthcare) | T1WI-Field of view (mm2 ):260 × 320; Slice Thickness (mm): 3.4; Echo time (ms):1.3;Repetition time (ms): 3.3; T2WI-Field of view (mm2 ):240 × 320; Slice Thickness (mm): 4.5; Echo time (ms):80;Repetition time (ms): 1600; DWI-Field of view (mm2 ):216 × 268; Slice Thickness (mm): 5; Echo time (ms):43;Repetition time (ms): 43; |
|  |  | 29 | 4 | 8 | 31 | 79.00% | 88.20% | MRI (T1WI model) | LASSO | 960 | NR | NR |  |  |
|  |  | 30 | 4 | 7 | 31 | 81.60% | 88.00% | MRI (T2WI model) | LASSO | 960 | NR | NR |  |  |
|  |  | 26 | 5 | 11 | 30 | 71.10% | 85.30% | MRI (DWI model) | LASSO | 960 | NR | NR |  |  |
|  |  | 32 | 6 | 5 | 29 | 85.70% | 82.40% | MRI (ADC model) | LASSO | 960 | NR | NR |  |  |
|  |  | 34 | 6 | 3 | 29 | 92.10% | 82.40% | MRI (Mp-MRI model) | LASSO | 960 | NR | NR |  |  |
|  |  | 35 | 4 | 2 | 31 | 93.90% | 87.50% | MRI (Mix-V model) | LASSO | 960 | NR | NR |  |  |
|  |  | 29 | 4 | 8 | 31 | 71.40% | 87.50% | MRI (T1WI-V model) | LASSO | 960 | NR | NR |  |  |
|  |  | 30 | 4 | 7 | 31 | 78.60% | 87.90% | MRI (T2WI-V model) | LASSO | 960 | NR | NR |  |  |
|  |  | 31 | 11 | 6 | 24 | 83.70% | 68.80% | MRI (DWI-V model) | LASSO | 960 | NR | NR |  |  |
|  |  | 33 | 4 | 4 | 31 | 89.50% | 87.50% | MRI (ADC-V model) | LASSO | 960 | NR | NR |  |  |
|  |  | 34 | 5 | 3 | 30 | 92.80% | 85.70% | MRI (Mp-MRI-V model) | LASSO | 960 | NR | NR |  |  |
| Ma | 2022 | 107 | 1 | 44 | 23 | 70.90% | 95.80% | CT (AP model) | LASSO | 1037 | NR | NR | Abdominal CT examination of 175 patients was performed using Discovery CT 750 HD (GE Healthcare, Milwaukee, WI, USA), Revolution CT (GE Healthcare), and 64-slice GE Optima CT660 spiral CT (GE Healthcare, Tokyo, Japan). | The parameters for Discovery CT 750 HD and Revolution CT were as follows: tube voltage: 120 kV, automatic mA modulation, pitch 0.984, and rack rotation time 0.5 s/cycle. Layer thickness and layer spacing were 0.625 mm. An EZEM double-barreled high-pressure syringe was used to inject the contrast agent Ioverol (320 mgI/mL) at the flow rate of 3.5 mL/s and dose 1 mL/kg. 64-layer GE Optima CT660 spiral CT: tube voltage 80–120 kV, tube current 200–400 mA, pitch 0.984, frame rotation time 0.8 s/cycle, collimator width 0.625×64 mm. |
|  |  | 132 | 1 | 19 | 23 | 87.40% | 95.80% | CT (VP model) | LASSO | 1037 | NR | NR |  |  |
|  |  | 132 | 1 | 19 | 23 | 87.40% | 95.80% | CT (Radiomics model) | LASSO | 1037 | NR | NR |  |  |
|  |  | 143 | 2 | 8 | 22 | 94.70% | 91.70% | CT (COMB model) | LASSO | 1037 | NR | NR |  |  |
| Zhang | 2022 | 46 | 6 | 13 | 38 | 77.97% | 86.36% | CT-Radiomics model-TC | LASSO | 1409 | NR | NR | 256- and 320-slice multidetector-row CT scanners (256 slices: Brilliance-16P, Philips Healthcare; 320 slices: Aquilion ONE, Canon Medical Systems) | The CT scanning parameters were as follows: 120 kV, 150 effective mAs,collimation of 160 × 0.5 mm, a matrix of 350 × 350, and a gantry rotation time of 0.5 s. |
|  |  | 22 | 0 | 4 | 9 | 83.33% | 95.65% | CT-Radiomics model-VC | LASSO | 1409 | NR | NR |  |  |
|  |  | 70 | 6 | 8 | 19 | 89.83% | 75.00% | CT-CT model-TC | LASSO | 1409 | NR | NR |  |  |
|  |  | 24 | 0 | 2 | 9 | 91.67% | 100.00% | CT-CT model-VC | LASSO | 1409 | NR | NR |  |  |
| Lu | 2023 | 45 | 0 | 0 | 22 | 100.00% | 100.00% | CT-RF | RF | 158 | NR | NR | 64-slice multidetector CT  (SOMATOM, Definition AS+, Siemens,Forchheim, Germany). | The parameters involved were as follows: 120 kVp; effective 180 mA; rotation time, 0.5 s; detector collimation, 32 × 1.2 mm; field of view, 350 × 350 mm; matrix, 512 × 512; section thickness, 5 mm; and reconstruction section thickness, 1.5 mm. |
|  |  | 43 | 1 | 2 | 21 | 95.00% | 97.00% | CT-MLR | MLR | 158 | NR | NR |  |  |
|  |  | 45 | 2 | 0 | 20 | 100.00% | 92.00% | CT-SVM | SVM | 158 | NR | NR |  |  |
|  |  | 43 | 2 | 2 | 20 | 95.00% | 90.00% | CT-DT | DT | 158 | NR | NR |  |  |
|  |  | 17 | 1 | 2 | 9 | 89.00% | 93.00% | CT-MLR | MLR | 158 | NR | NR |  |  |
|  |  | 18 | 5 | 1 | 5 | 93.00% | 48.00% | CT-SVM | SVM | 158 | NR | NR |  |  |
|  |  | 17 | 3 | 2 | 7 | 87.00% | 72.00% | CT-DT | DT | 158 | NR | NR |  |  |
|  |  | 17 | 8 | 2 | 2 | 100.00% | 17.00% | CT-RF | RF | 158 | NR | NR |  |  |
| Malagi | 2023 | 21 | 1 | 4 | 5 | 83.00% | 76.00% | MRI | ANNs | NR | NR | NR | MRI on a 1.5T scanner (Achieva, Philips Healthcare, Best, The Netherlands) using a multi-channel phased-array body coil. | The MRI sequence protocol used to acquire all the patients’ scans included gradient echo T1-weighted (mDixon) with repetition time (TR): 500 ms and echo time (TE): 2.3 and 4.6 ms, fat-suppressed (FS) T2-weighted (turbo spin-echo (TSE)) was acquired in the axial plane with TR: 1000 ms  and TE: 80 ms. Steady-state free precession (SSFP) sequences were acquired in the coro-nal plane with TR: 500 ms and TE: 50 ms. Thick slab half-Fourier acquisition single-shot turbo spin echo (HASTE) with TR: 8000 ms and TE: 800 ms and driven equilibrium with 90-degree flip-back pulse (RESTORE) with TR: 1000 ms and TE: 6500 ms sequences for heavily T2-weighted Thick slab magnetic resonance cholangiopancreatography sequence (MRCP) were performed. |
| Qu | 2023 | 21 | 3 | 5 | 13 | 80.72% | 81.25% | CT | LASSO | 174 | NR | NR | multidetector CT scanners (Discovery CT750 HD, General Electric Company, United States) | slice thickness, 0.625 mm; reconstruction slice thickness/interval, 1.25 mm; tube voltage, 120 kV; table speed, 39.37 mm/rotation; rotation time, 0.5 s; detector pitch, 0.984:1; matrix, 512*512. |
| Nakamura | 2024 | 53 | 9 | 8 | 15 | 87.00% | 63.00% | EUS | CNNs | NR | NR | NR | Prosound F75 ultrasound system (Hitachi Aloka Med  ical) with a GF-UCT260 curved linear echoendoscope (Olympus Corporation). | Before inserting a biopsy needle, endoscopists observed the lesion and recorded a video clip for approximately 10 s. |
| Zhang | 2025 | 47 | 8 | 8 | 47 | 86.00% | 86.00% | EUS | LASSO | NR | NR | NR | Ultrasound examinations were performed using Philips IU22,  Philips EPIQ5, SIEMENS S3000, and Mindray DC-8 color Doppler ultrasound diagnostic instruments and an abdominal convex array probe with a frequency of 1.0–6.0 MHz. | NR |
| Zhang | 2025 | 47 | 11 | 8 | 44 | 86.00% | 80.00% | EUS | LASSO | NR | NR | NR |  |  |
| Zhang | 2025 | 40 | 3 | 15 | 52 | 72.00% | 94.00% | EUS | LASSO | NR | NR | NR |  |  |

**Table S3:** Detail characteristics of the 25 included studies with 25 best diagnostic performance tables.

| **Author** | **Year** | **Total** | **TP** | **FP** | **FN** | **TN** | **Sensitivity** | **Specificity** | **Algorithm architecture** |
| --- | --- | --- | --- | --- | --- | --- | --- | --- | --- |
| Norton | 2001 | 35 | 19 | 3 | 2 | 11 | 89.00% | 79.00% | ANNs |
| Adrian | 2008 | 43 | 30 | 4 | 2 | 7 | 93.80% | 63.60% | ANNs |
| Adrian | 2012 | 258 | 185 | 8 | 26 | 39 | 87.59% | 82.94% | ANNs |
| Zhu | 2013 | 388 | 242 | 9 | 20 | 117 | 92.52% | 93.03% | SVM |
| Adrian | 2015 | 167 | 98 | 4 | 14 | 51 | 87.50% | 92.72% | ANNs |
| Zhang | 2019 | 110 | 59 | 5 | 6 | 40 | 89.24% | 89.33% | SVM |
| Ren | 2019 | 109 | 74 | 2 | 5 | 28 | 94.00% | 92.00% | TFBs |
| Park | 2020 | 62 | 26 | 0 | 3 | 33 | 89.70% | 100.00% | RF |
| Lin | 2020 | 96 | 48 | 2 | 3 | 43 | 93.30% | 96.10% | RF |
| Marya | 2020 | 438 | 266 | 10 | 26 | 136 | 91.00% | 93.00% | CNNs |
| Ziegelmayer | 2020 | 86 | 37 | 7 | 5 | 37 | 89.00% | 83.00% | CNNs |
| Ren | 2020 | 109 | 73 | 2 | 6 | 28 | 92.20% | 94.20% | RF |
| Liu | 2021 | 112 | 55 | 2 | 9 | 46 | 85.31% | 96.04% | SVM-RFE |
| Li | 2021 | 97 | 52 | 3 | 3 | 39 | 95.24% | 92.73% | LASSO |
| Deng | 2021 | 64 | 50 | 0 | 1 | 13 | 98.00% | 100.00% | SVM |
| Tong | 2022 | 109 | 71 | 6 | 2 | 30 | 97.30% | 83.30% | DLR |
| Zhang | 2022 | 111 | 57 | 7 | 9 | 38 | 86.00% | 84.00% | SVM-RFE |
| Anai | 2022 | 50 | 30 | 5 | 0 | 15 | 100.00% | 75.00% | SVM |
| Wei | 2022 | 112 | 56 | 3 | 8 | 45 | 87.50% | 93.00% | MF |
| Shiraishi | 2022 | 105 | 47 | 1 | 30 | 27 | 60.70% | 96.10% | SVM |
| Liu | 2022 | 72 | 34 | 5 | 3 | 30 | 92.20% | 87.10% | LASSO |
| Ma | 2022 | 175 | 145 | 2 | 8 | 22 | 94.70% | 91.70% | LASSO |
| Zhang | 2022 | 103 | 46 | 6 | 13 | 38 | 77.97% | 86.36% | LASSO |
| Lu | 2023 | 67 | 45 | 0 | 0 | 22 | 100.00% | 100.00% | RF |
| Malagi | 2023 | 31 | 21 | 1 | 4 | 5 | 83.00% | 76.00% | ANNs |
| Qu | 2023 | 42 | 21 | 3 | 5 | 13 | 80.72% | 81.25% | LASSO |
| Nakamura | 2024 | 85 | 53 | 9 | 8 | 15 | 87.00% | 63.00% | CNNs |
| Zhang | 2025 | 110 | 47 | 8 | 8 | 47 | 86.00% | 86.00% | LASSO |

**Table S4:** The pooled sensitivity, specificity and AUC of different groups.

| **Group** | **No. of study** | **No. of table** | **Sensitivity** | **Specificity** | **PLR** | **NLR** | **DOR** | **AUC** |
| --- | --- | --- | --- | --- | --- | --- | --- | --- |
| **28 study with 76 tables** | 28 | 76 | 0.89[0.87-0.90] | 0.88[0.86-0.90] | 7.5[6.2-9.0] | 0.13[0.11-0.15] | 58[43-76] | 0.94[0.92-0.96] |
| **28 study with 28 tables** | 28 | 28 | 0.91[0.88-0.93] | 0.90[0.87-0.93] | 9.2[6.9-12.3] | 0.10[0.08-0.13] | 92[60-142] | 0.96[0.94-0.97] |
| **Clinician** | 8 | 14 | 0.77[0.66-0.85] | 0.80[0.71-0.87] | 3.9[2.6-5.6] | 0.29[0.20-0.42] | 13[7-23] | 0.85[0.82-0.88] |
| **AI in 8 study with Clinician** | 8 | 32 | 0.88[0.85-0.91] | 0.88[0.85-0.90] | 7.2[5.8-8.8] | 0.13[0.10-0.17] | 54[37-79] | 0.93[0.91-0.95] |

**Table S5: The sensitivity analysis results of different groups.**

| **Author** | **Year** | **Sensitivity** | I2 **(%)** | **Specifificity** | I2 **(%)** | **DOR** | **AUROC** |
| --- | --- | --- | --- | --- | --- | --- | --- |
| Norton | 2001 | 0.89 [0.87, 0.90] | 77.43 | 0.88 [0.86, 0.90] | 75.96 | 58[44-77] | 0.94[0.92-0.96] |
| Adrian | 2008 | 0.88 [0.87, 0.90] | 77.32 | 0.88 [0.86, 0.90] | 75.67 | 58[44-77] | 0.94[0.92-0.96] |
| Adrian | 2012 | 0.89 [0.87, 0.90] | 77.42 | 0.88 [0.86, 0.90] | 76.03 | 58[44-77] | 0.94[0.92-0.96] |
| Zhu | 2013 | 0.88 [0.86, 0.90] | 76.82 | 0.88 [0.85, 0.90] | 74.78 | 55[42-73] | 0.94[0.92-0.96] |
| Adrian | 2015 | 0.89 [0.87, 0.90] | 77.47 | 0.88 [0.86, 0.90] | 75.68 | 57[43-76] | 0.94[0.92-0.96] |
| Zhang | 2019 | 0.89 [0.87, 0.90] | 77.33 | 0.88 [0.86, 0.90] | 75.91 | 57[43-76] | 0.94[0.92-0.96] |
| Ren | 2019 | 0.89 [0.87, 0.90] | 76.71 | 0.88 [0.86, 0.90] | 76.54 | 57[43-76] | 0.94[0.92-0.96] |
| Park | 2020 | 0.89 [0.87, 0.90] | 77.30 | 0.88 [0.85, 0.90] | 75.20 | 56[42-74] | 0.94[0.92-0.96] |
| Lin | 2020 | 0.89 [0.87, 0.91] | 73.66 | 0.88 [0.85, 0.90] | 75.99 | 58[43-77] | 0.94[0.92-0.96] |
| Marya | 2020 | 0.88 [0.86, 0.90] | 77.09 | 0.88 [0.85, 0.90] | 76.19 | 56[42-76] | 0.94[0.92-0.96] |
| Ziegelmayer | 2020 | 0.89 [0.87, 0.90] | 77.46 | 0.88 [0.86, 0.90] | 76.02 | 58[44-77] | 0.94[0.92-0.96] |
| Ren | 2020 | 0.89 [0.87, 0.90] | 77.47 | 0.88 [0.86, 0.90] | 76.08 | 58[43-77] | 0.94[0.92-0.96] |
| Liu | 2021 | 0.89 [0.87, 0.91] | 77.99 | 0.88 [0.86, 0.90] | 60.22 | 60[47-78] | 0.95[0.92-0.96] |
| Li | 2021 | 0.88 [0.86, 0.90] | 77.07 | 0.88 [0.86, 0.90] | 75.67 | 56[43-75] | 0.94[0.92-0.96] |
| Deng | 2021 | 0.88 [0.86, 0.90] | 76.45 | 0.88 [0.86, 0.90] | 76.08 | 54[41-72] | 0.94[0.92-0.96] |
| Tong | 2022 | 0.88 [0.86, 0.90] | 77.02 | 0.88 [0.86, 0.90] | 76.27 | 56[42-75] | 0.94[0.92-0.96] |
| Zhang | 2022 | 0.89 [0.87, 0.90] | 77.48 | 0.88 [0.86, 0.90] | 76.01 | 58[44-77] | 0.94[0.92-0.96] |
| Anai | 2022 | 0.88 [0.86, 0.90] | 76.99 | 0.88 [0.86, 0.90] | 75.85 | 57[43-76] | 0.94[0.92-0.96] |
| Wei | 2022 | 0.89 [0.87, 0.91] | 77.63 | 0.88 [0.86, 0.90] | 76.33 | 59[44-78] | 0.95[0.92-0.96] |
| Shiraishi | 2022 | 0.89 [0.87, 0.90] | 73.89 | 0.88 [0.86, 0.90] | 75.56 | 58[44-77] | 0.94[0.92-0.96] |
| Liu | 2022 | 0.89 [0.87, 0.90] | 80.01 | 0.89 [0.86, 0.91] | 79.31 | 64[46-89] | 0.95[0.93-0.96] |
| Ma | 2022 | 0.89 [0.87, 0.90] | 75.05 | 0.88 [0.85, 0.90] | 75.98 | 56[42-75] | 0.94[0.92-0.96] |
| Zhang | 2022 | 0.89 [0.87, 0.90] | 78.00 | 0.88 [0.86, 0.90] | 76.46 | 58[43-77] | 0.94[0.92-0.96] |
| Lu | 2023 | 0.88 [0.86, 0.89] | 77.22 | 0.88 [0.86, 0.90] | 72.43 | 54[41-71] | 0.94[0.92-0.96] |
| Malagi | 2023 | 0.89 [0.87, 0.90] | 77.48 | 0.88 [0.86, 0.90] | 75.99 | 58[44-77] | 0.94[0.92-0.96] |
| Qu | 2023 | 0.89 [0.87, 0.90] | 77.48 | 0.88 [0.86, 0.90] | 76.02 | 58[44-77] | 0.95[0.92-0.96] |
| Nakamura | 2024 | 0.89 [0.87, 0.90] | 77.46 | 0.88 [0.86, 0.90] | 75.23 | 59[44-78] | 0.95[0.92-0.96] |
| Zhang | 2025 | 0.89 [0.87, 0.91] | 77.66 | 0.88 [0.86, 0.90] | 76.19 | 59[44-79] | 0.95[0.92-0.96] |

**Fig S1:** Supplementary Figure 1. QUADAS-2 summary plot.

**
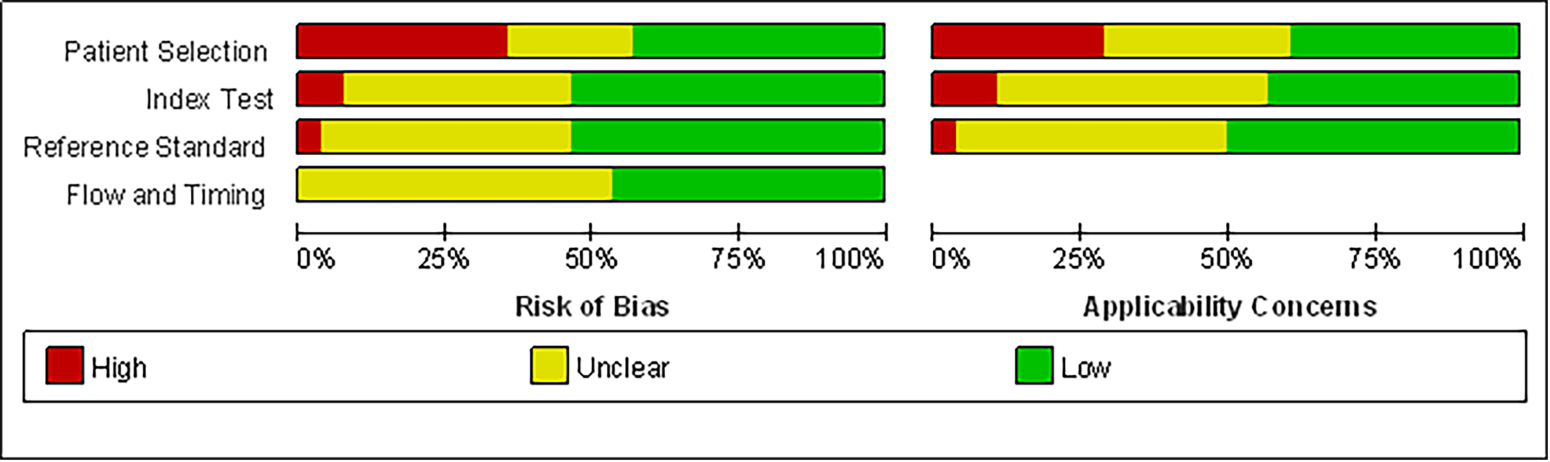
**

**Fig S2:** Risk of bias and concern of applicability for each item in included.


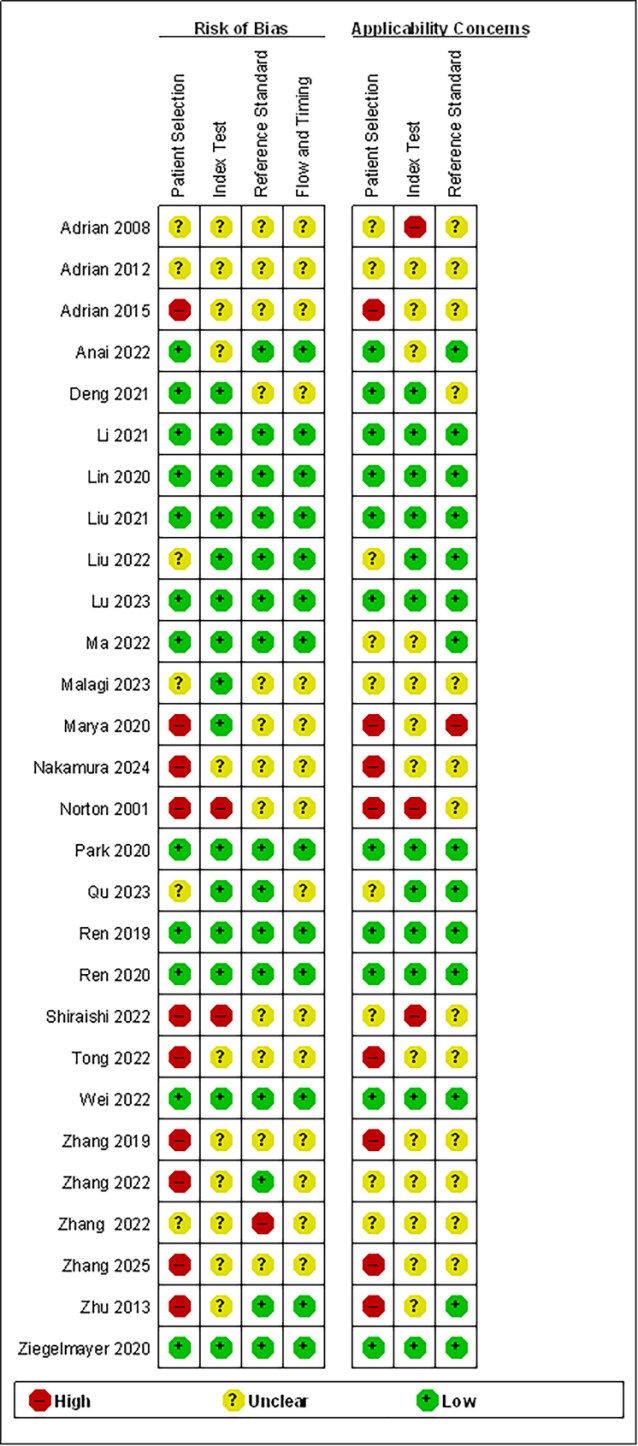


**
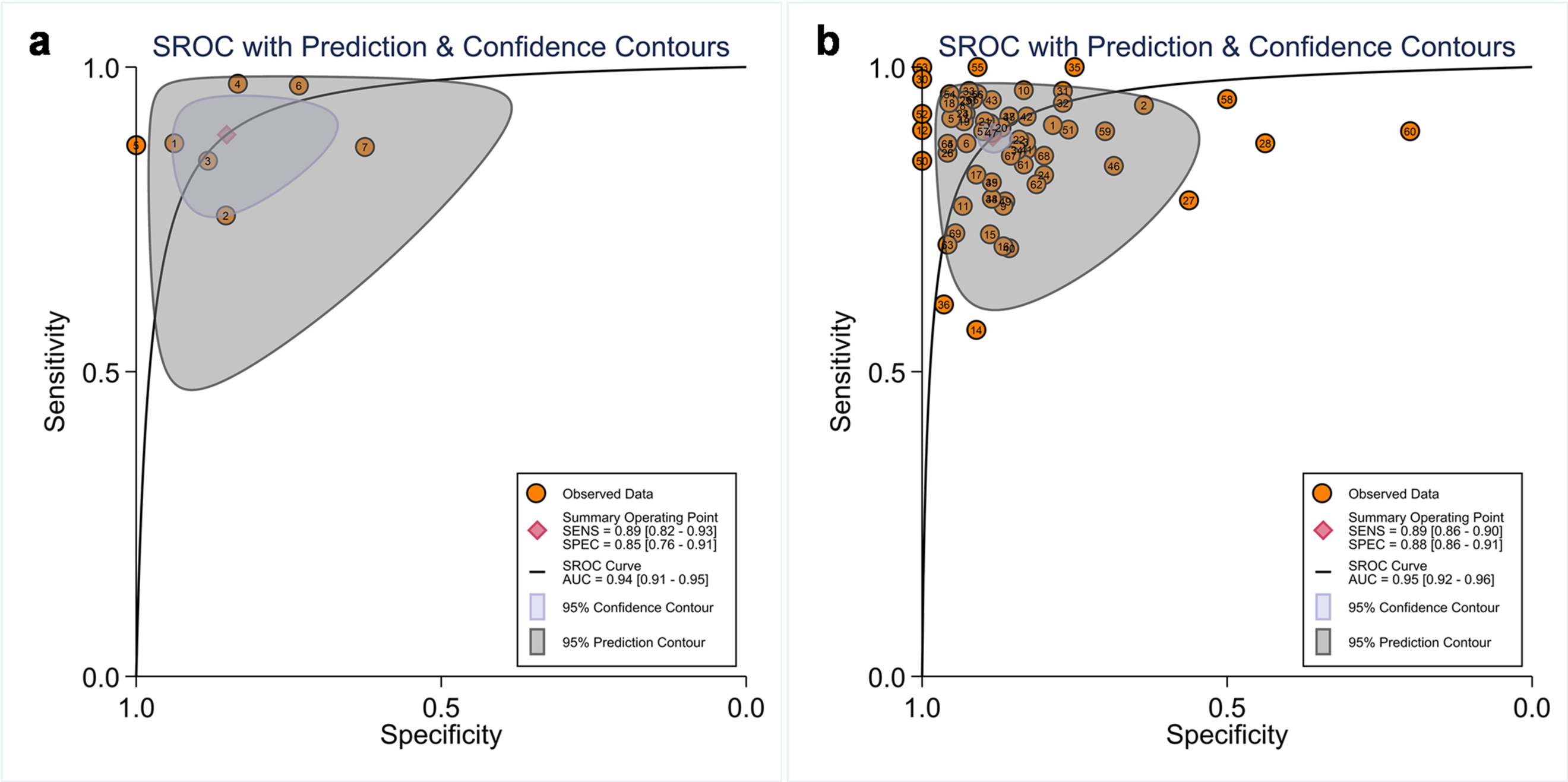
**

**Fig S3:** SROC curve of studies using different algorithms (DL or ML).

**a:** DL algorithms (3 studies with 7 tables)

**b:** ML algorithms (25 studies with 69 tables)

Abbreviations: DL: deep learning; ML: machine learning; SROC=summary receiver operating characteristic; SENS=summary sensitivity; SPEC=summary specificity.

**
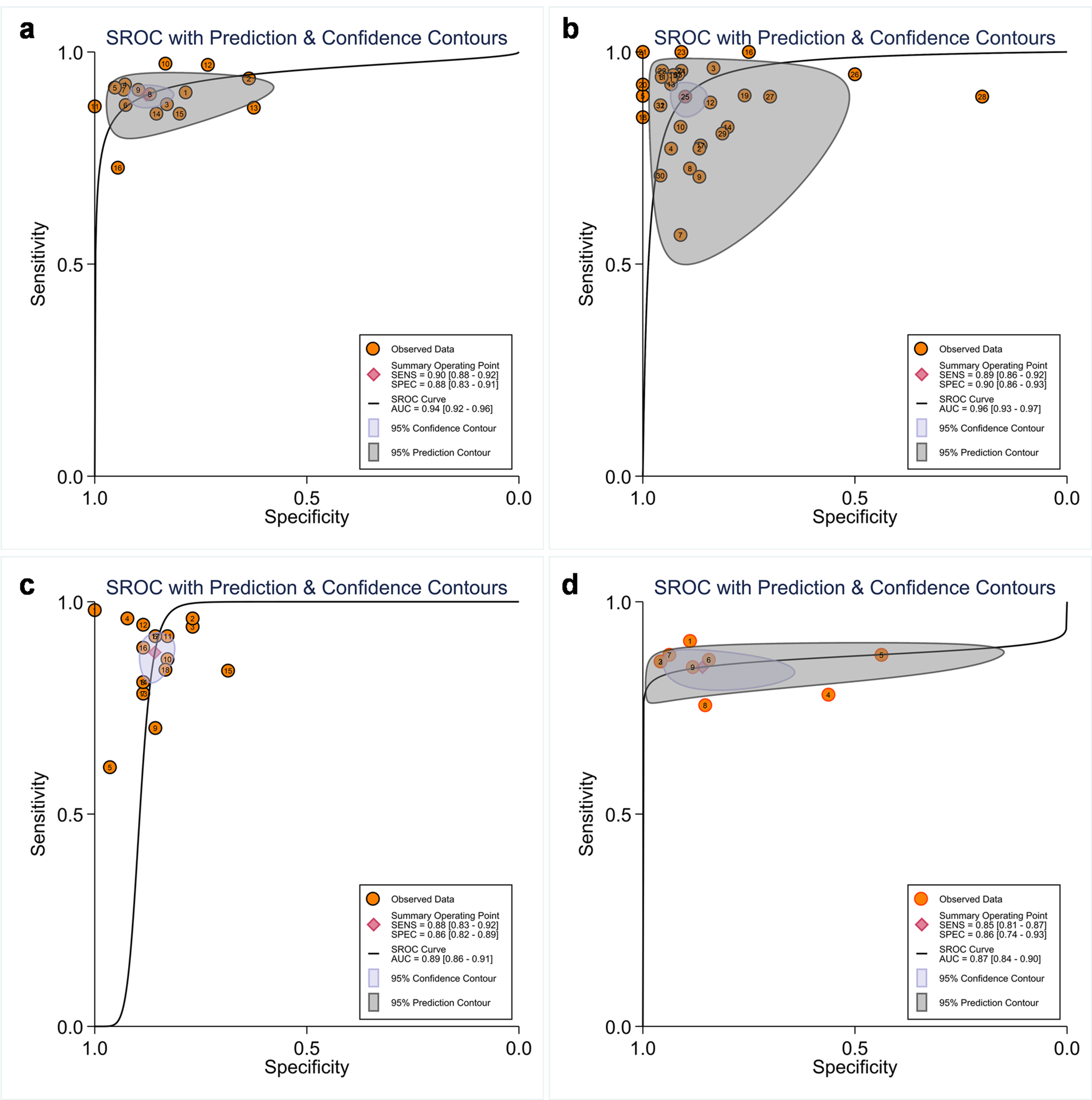
**

**Fig S4: SROC curve of different imaging modalities (US, CT, MRI, or PET).**

**a:** US (9 studies with 16 tables)

**b:** CT (11 studies with 33 tables)

**c:** MRI (4 studies with 18 tables)

**d:** PET (4 studies with 9 tables)

Abbreviations: AI: artificial intelligence; US: ultrasound; CT: computed tomography; MRI: magnetic resonance imaging; PET: positron emission tomography; SROC=summary receiver operating characteristic; SENS=summary sensitivity; SPEC=summary specificity.

**
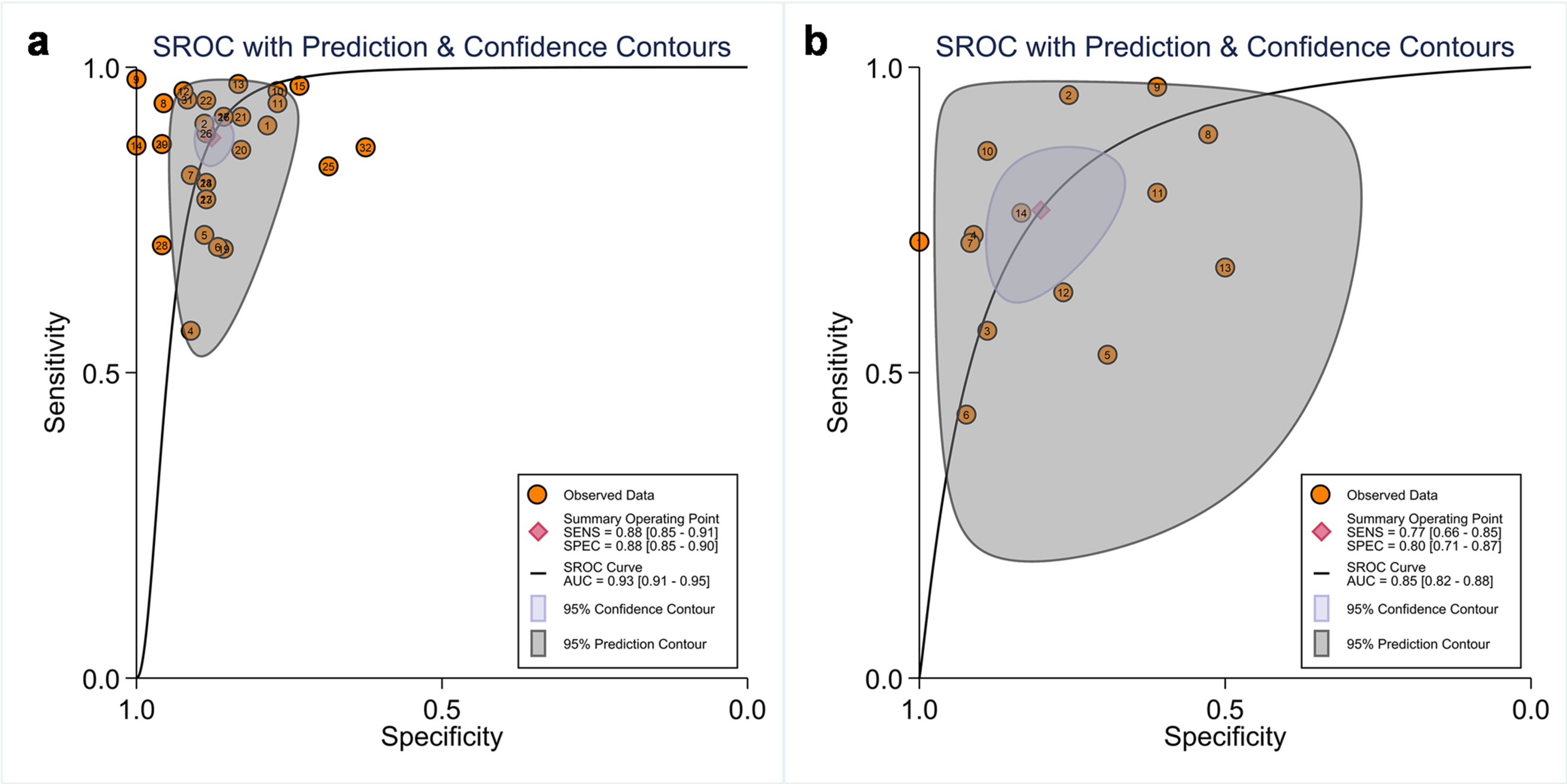
**

**Fig S5: SROC curve of AI vs** Clinician in same dataset**.**

**a:** AI (8 studies with 32 tables)

**b:** Clinician (8 studies with 14 tables)

**
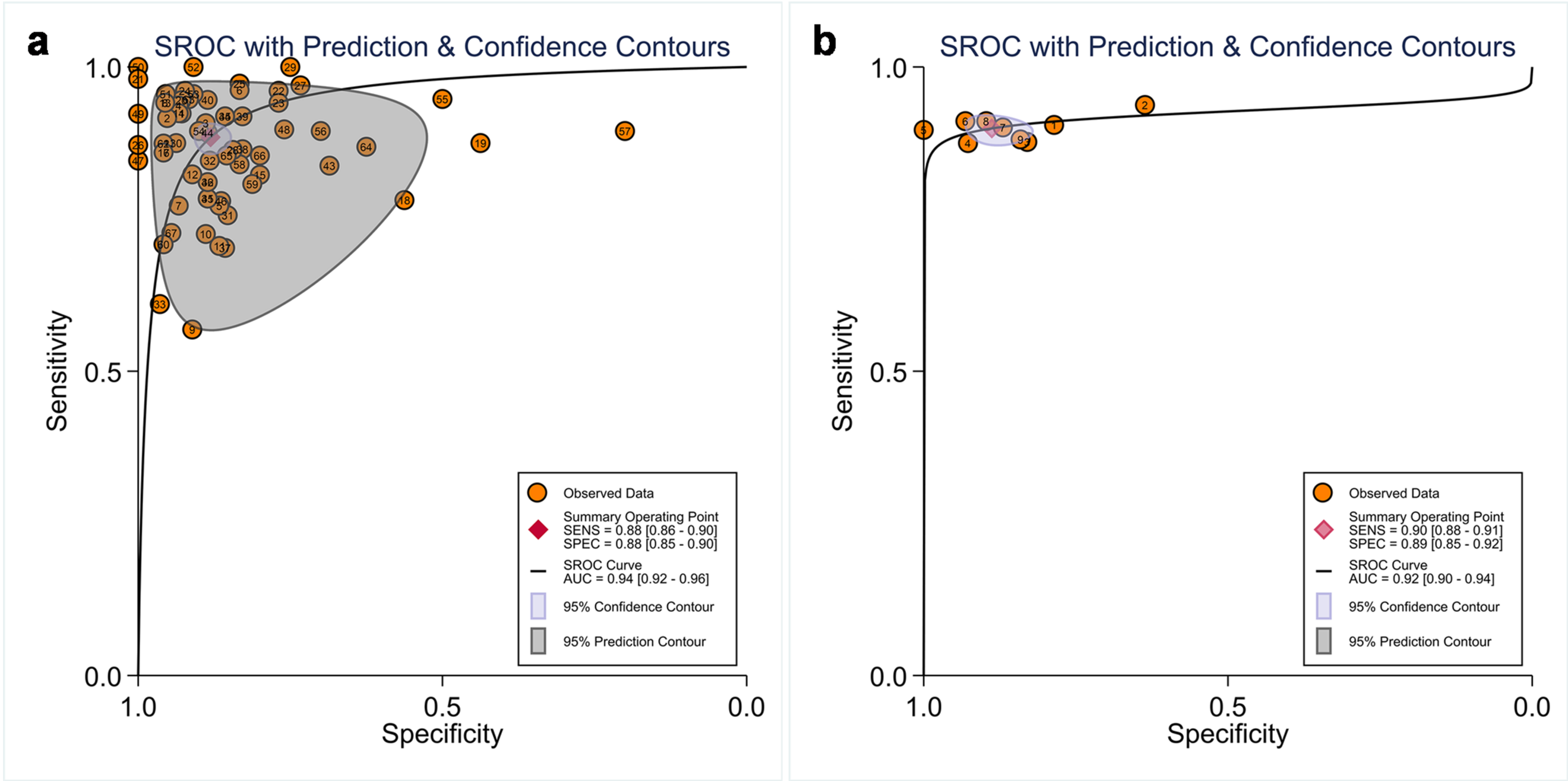
**

**Fig S6: SROC curve of different geographical distribution (Asia or non Asia).**

**a:** Geographical distribution in Asia (21 studies with 67 tables)

**b:** Geographical distribution in non-Asia (7 studies with 9 tables)

Abbreviations: AI: artificial intelligence; SROC=summary receiver operating characteristic; SENS=summary sensitivity; SPEC=summary specificity.

**
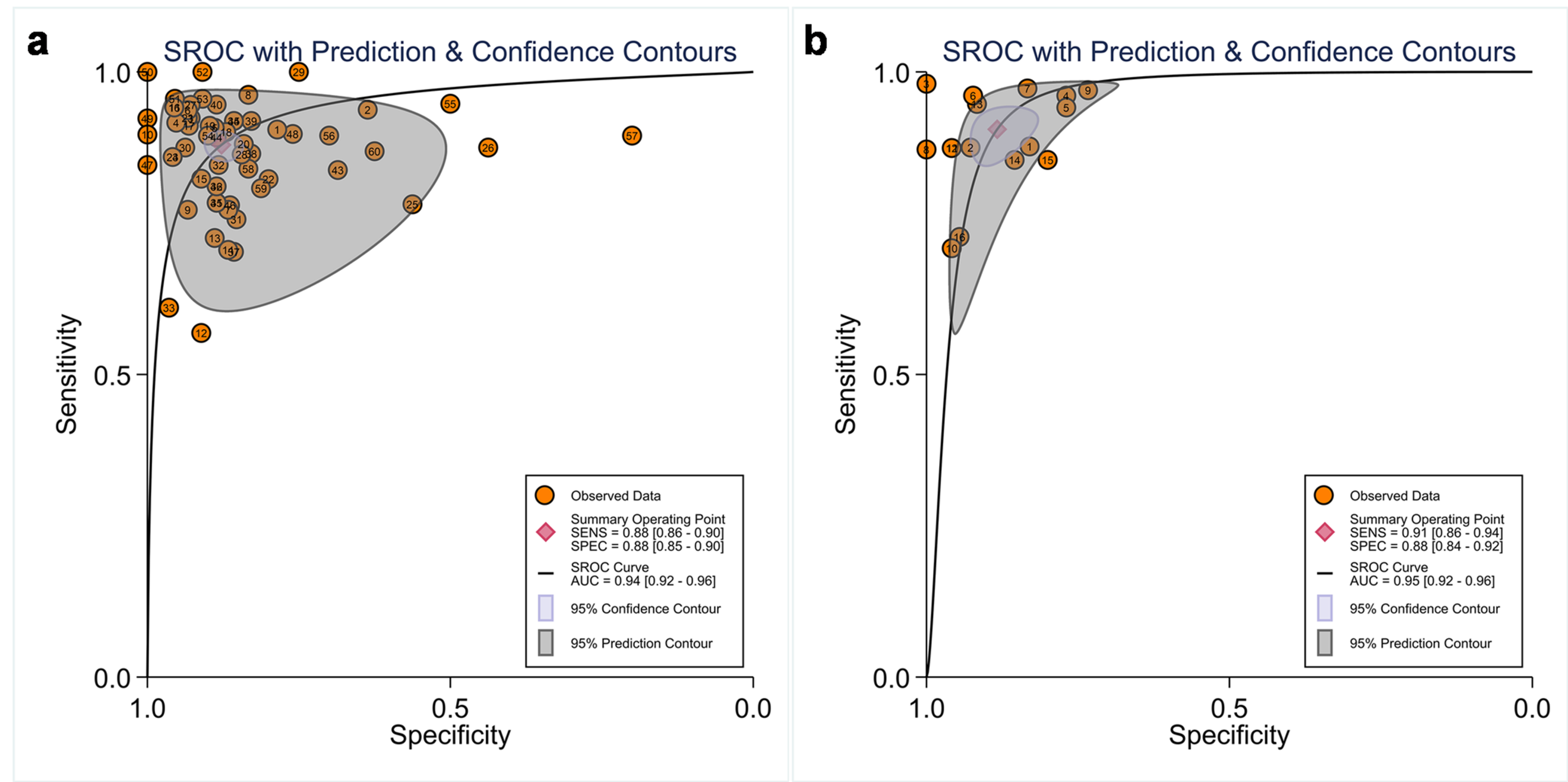
**

**Fig S7: SROC curve of different risk of bias levels (single or multiple).**

**a:** single (22 studies with 60 tables)

**b:** multi (6 studies with 16 tables)

Abbreviations: AI: artificial intelligence; SROC=summary receiver operating characteristic; SENS=summary sensitivity; SPEC=summary specificity.

**
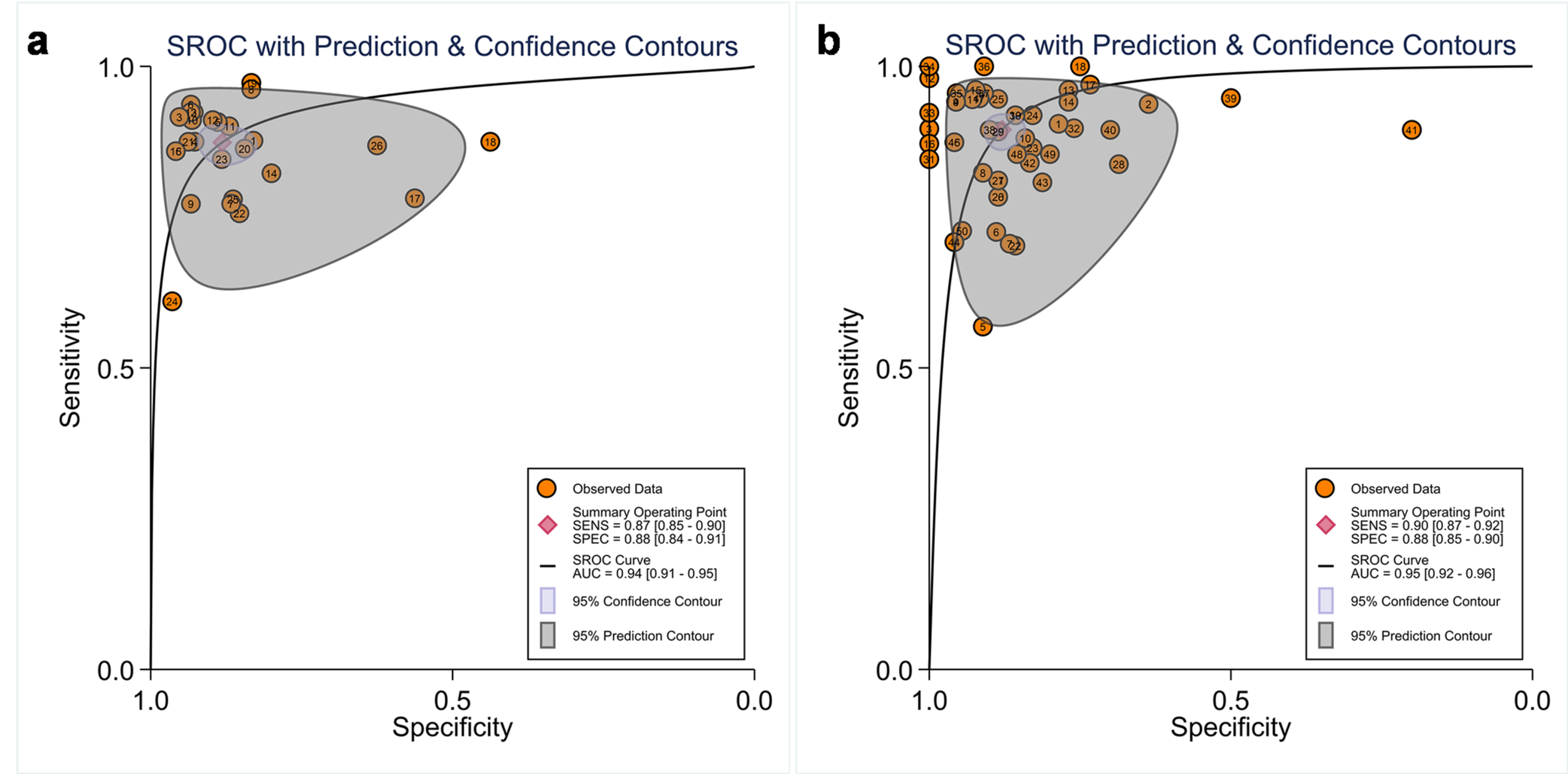
**

**Fig S8: SROC curve of studies with different sample size(≤ 100 or > 100).**

**a:** Sample size ≤ 100 (14 studies with 26 tables)

**b:** Sample size > 100 (14 studies with 50 tables)

Abbreviations: AI: artificial intelligence; ROC=receiver operating characteristic; SENS= sensitivity; SPEC= specificity.

**
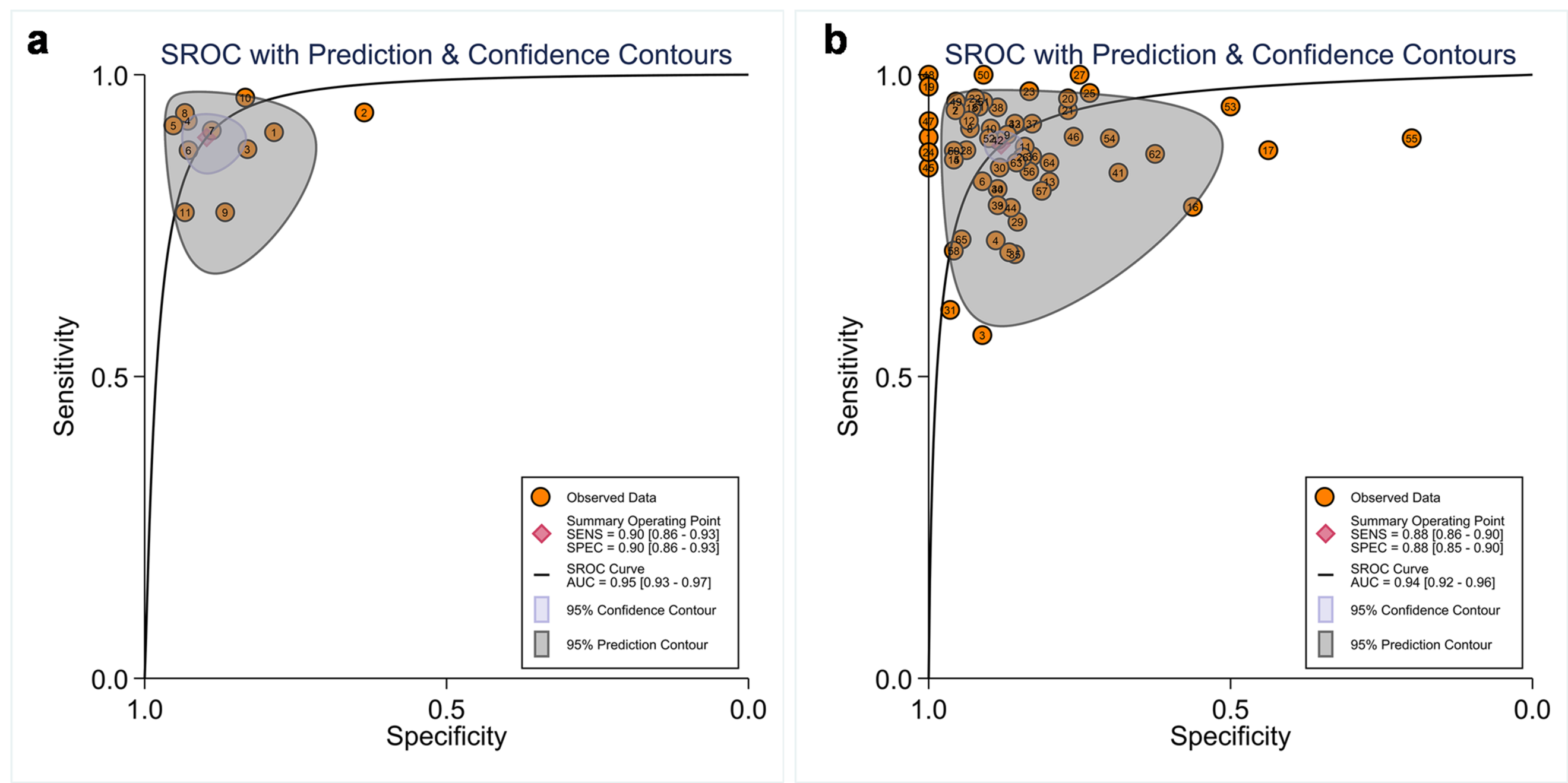
**

**Fig S9: SROC curve of different publication year (before or after 2020).**

**a:** Year of publication before 2020 (7 studies with 11 tables)

**b:** Year of publication after 2020 (21 studies with 65 tables)

Abbreviations: AI: artificial intelligence; SROC=summary receiver operating characteristic; SENS=summary sensitivity; SPEC=summary specificity.


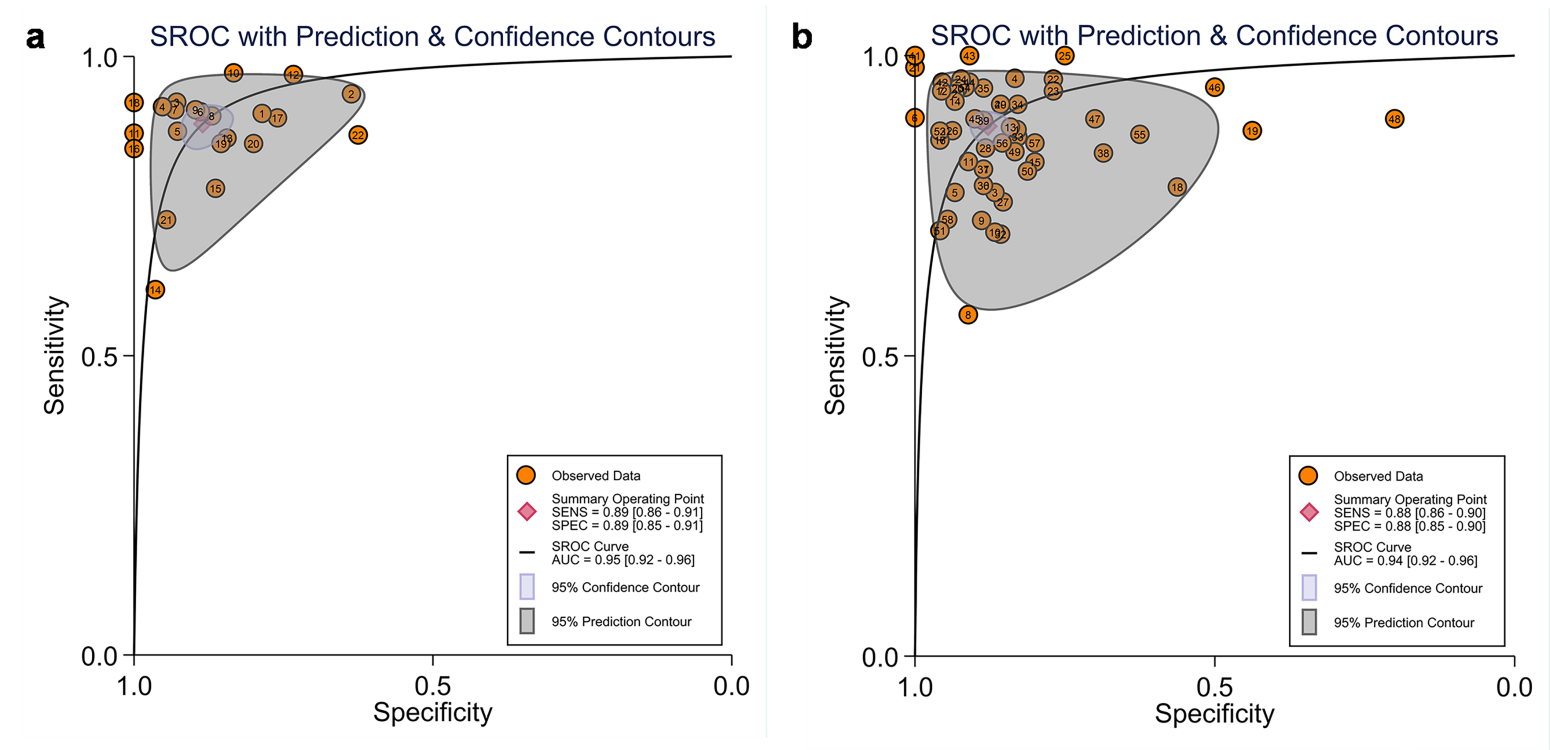


**Fig S10: SROC curve of different risk of bias levels (High/Unclear or Low)**

**a:** Low risk of bias and concern of applicability of study (9 studies with 30 tables)

**b:** High/Unclear risk of bias and concern of applicability of study (19 studies with 46 tables)

Abbreviations: AI: artificial intelligence; SROC=summary receiver operating characteristic; SENS=summary sensitivity; SPEC=summary specificity.


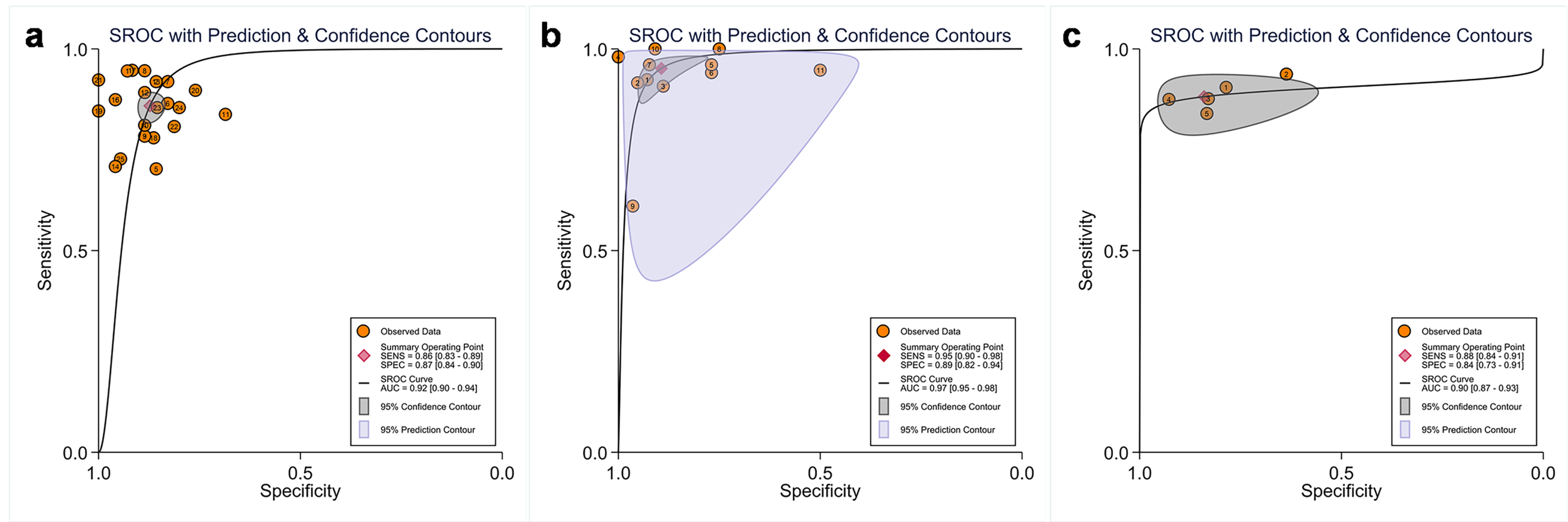


**Fig S11: SROC curve of** different AI algorithm**.**

**a:** Pooled AI algorithm of LASSO in this meta study. (6 studies with 25 tables)

**b:** Pooled AI algorithm of SVM in this meta study. (6 studies with 11 tables)

**c:** Pooled AI algorithm of ANNs in this meta study. (5 studies with 5 tables)

Abbreviations: AI: artificial intelligence; SROC=summary receiver operating characteristic; SENS=summary sensitivity; SPEC=summary specificity.

**
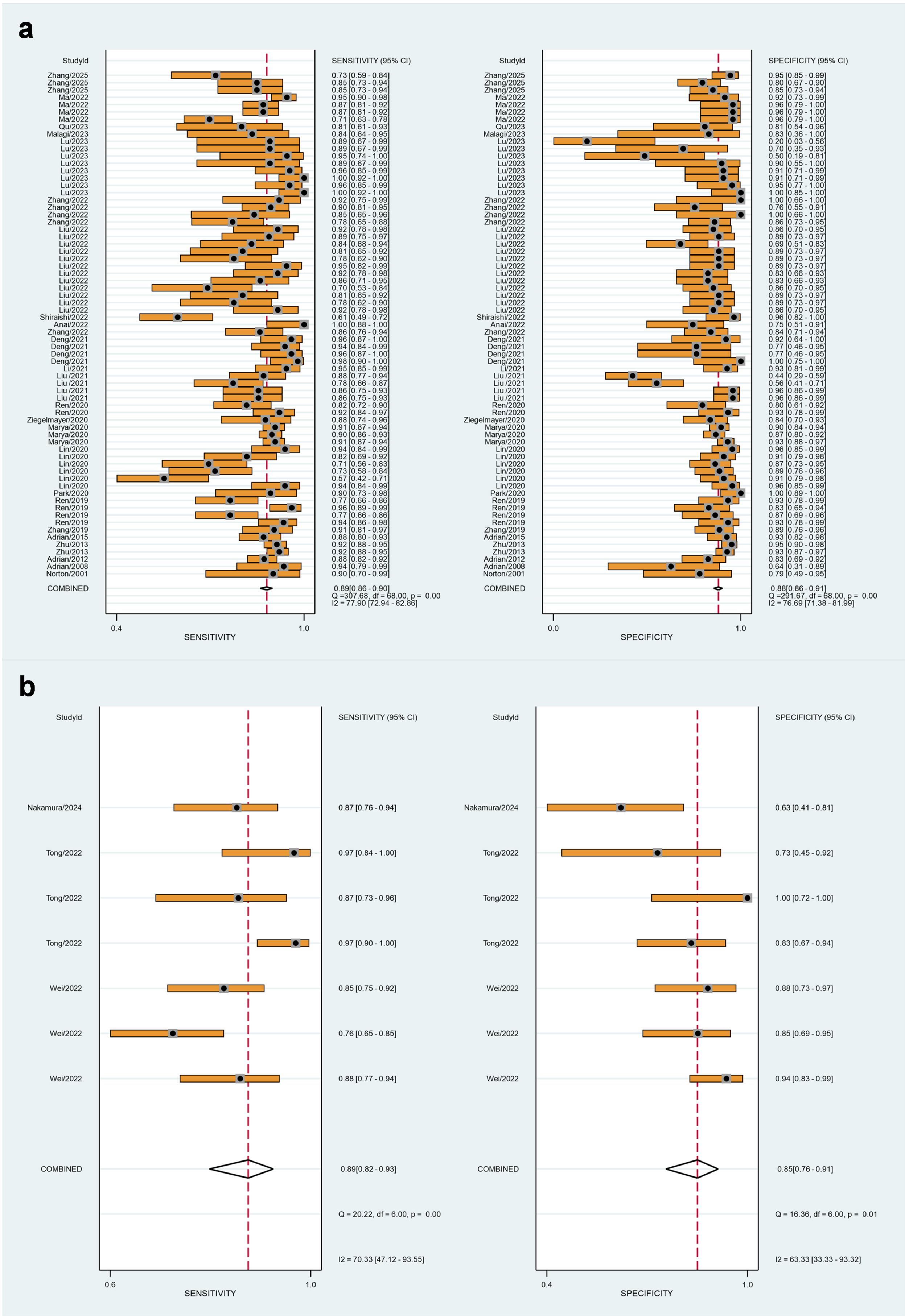
**

**Fig S12: Forest plot of different studies using different algorithms (DL or ML).**

**a:** ML algorithms (25 studies with 69 tables)

**b:** DL algorithms (3 studies with 7 tables)

**
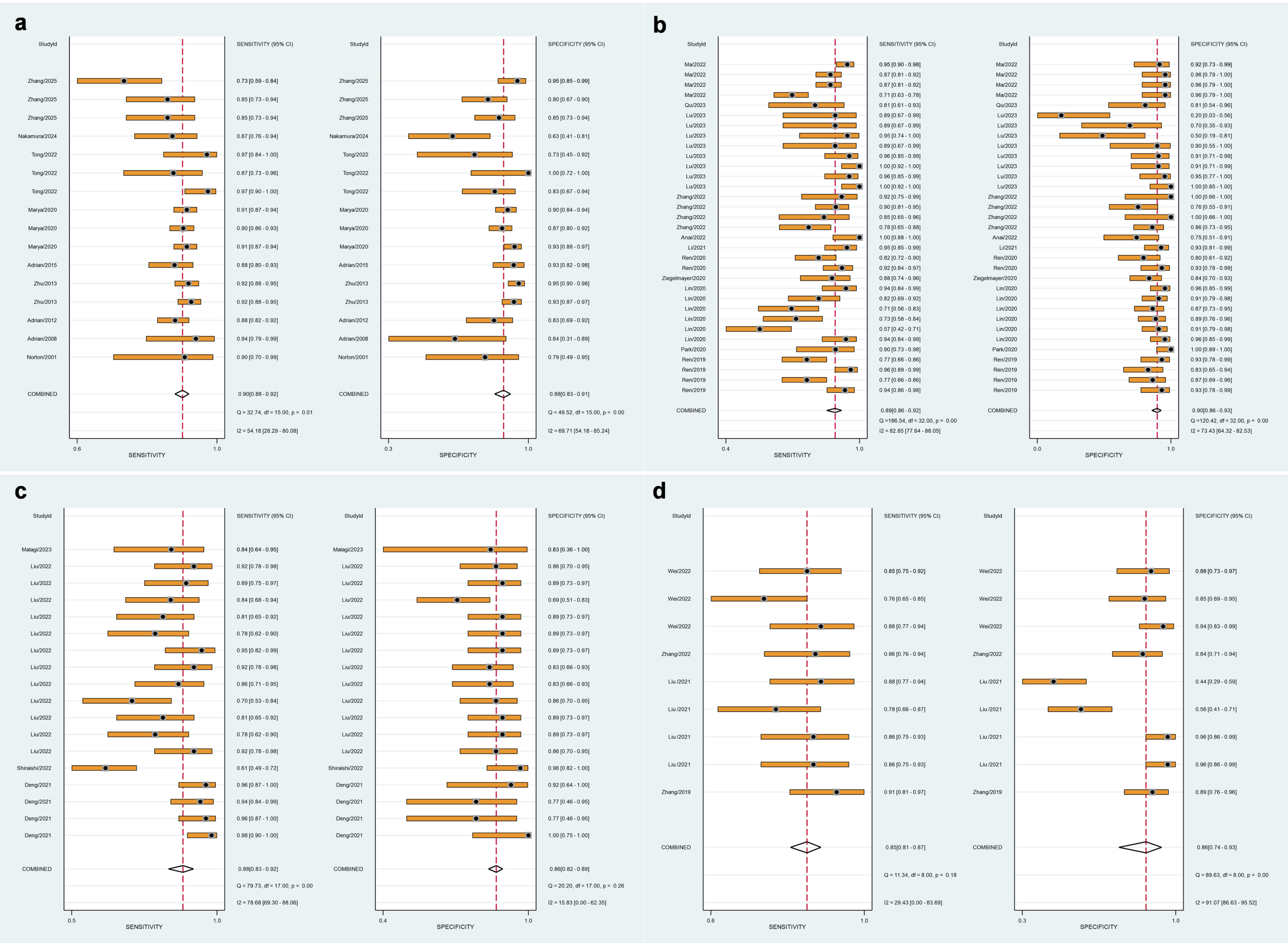
**

**Fig S13: Forest plot of different imaging modalities (US, CT, MRI, or PET).**

**a:** US (9 studies with 16 tables)

**b:** CT (11 studies with 33 tables)

**c:** MRI (4 studies with 18 tables)

**d:** PET (4 studies with 9 tables)

**
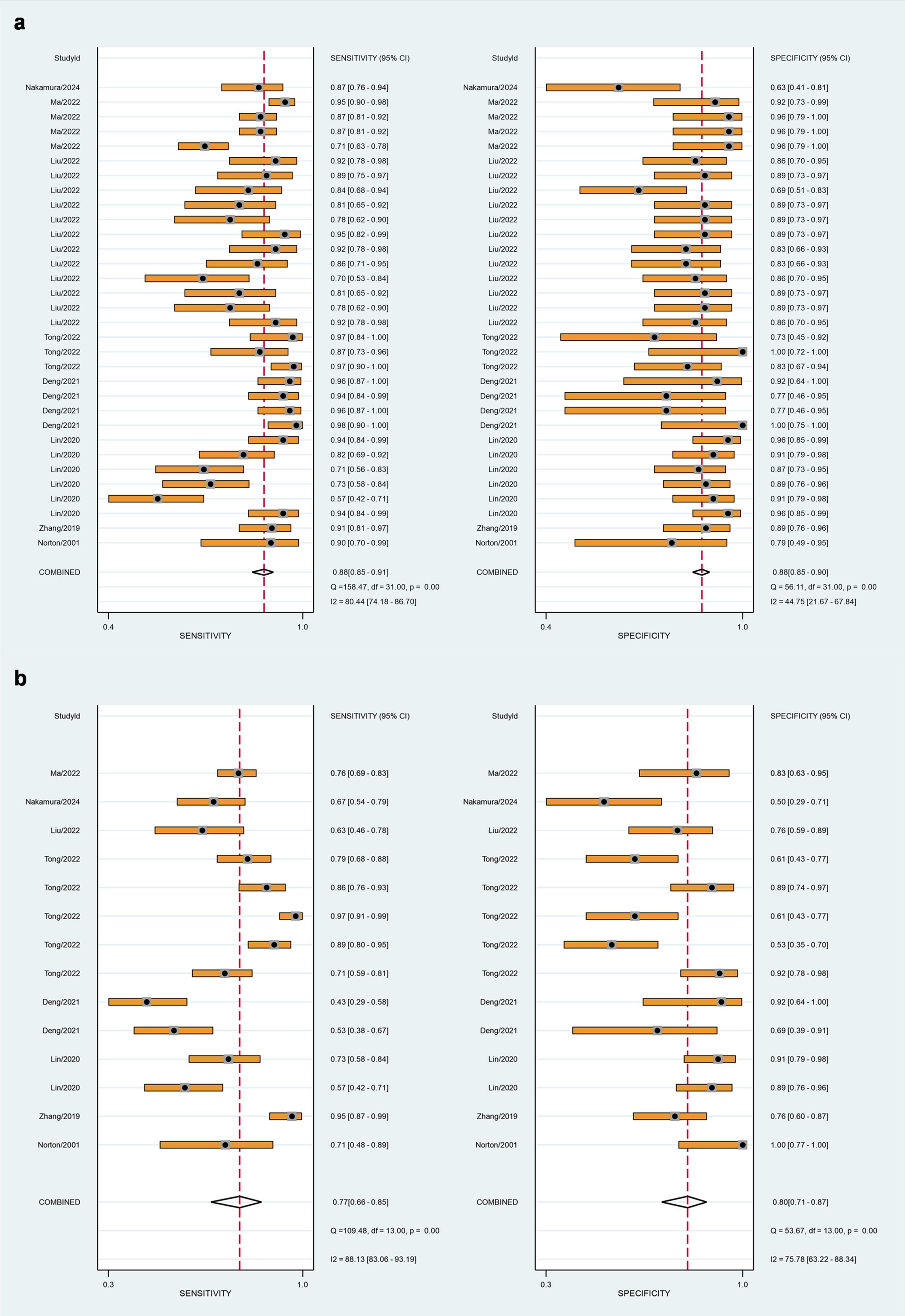
**

**Fig S14: Forest plot of AI vs Clinician in same dataset.**

**a:** AI (8 studies with 32 tables)

**b:** Clinician (8 studies with 14 tables)

**
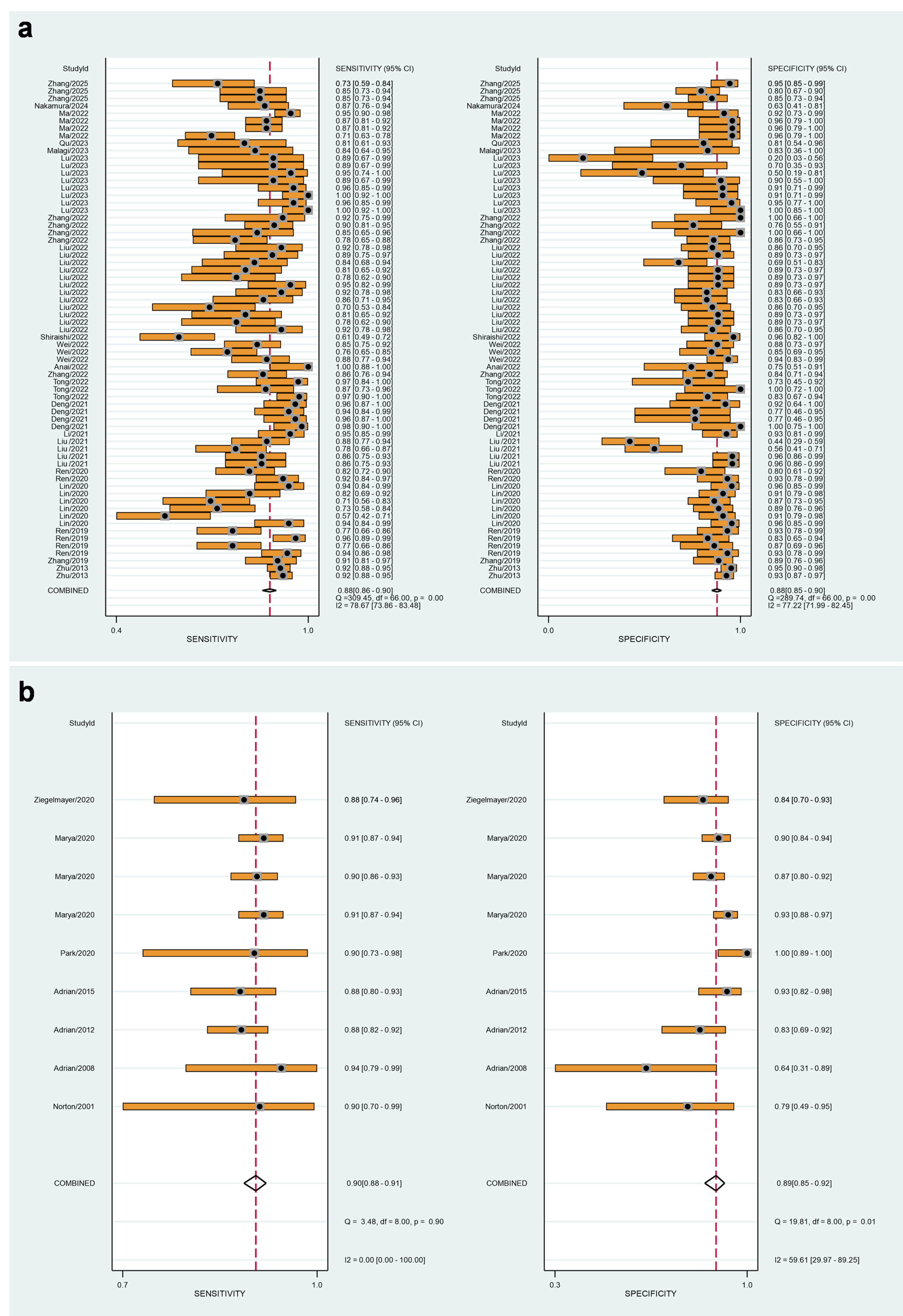
**

**Fig S15: Forest plot of different geographical distribution (Asia or non Asia).**

**a:** Geographical distribution in Asia (21 studies with 67 tables)

**b:** Geographical distribution in non-Asia (7 studies with 9 tables)


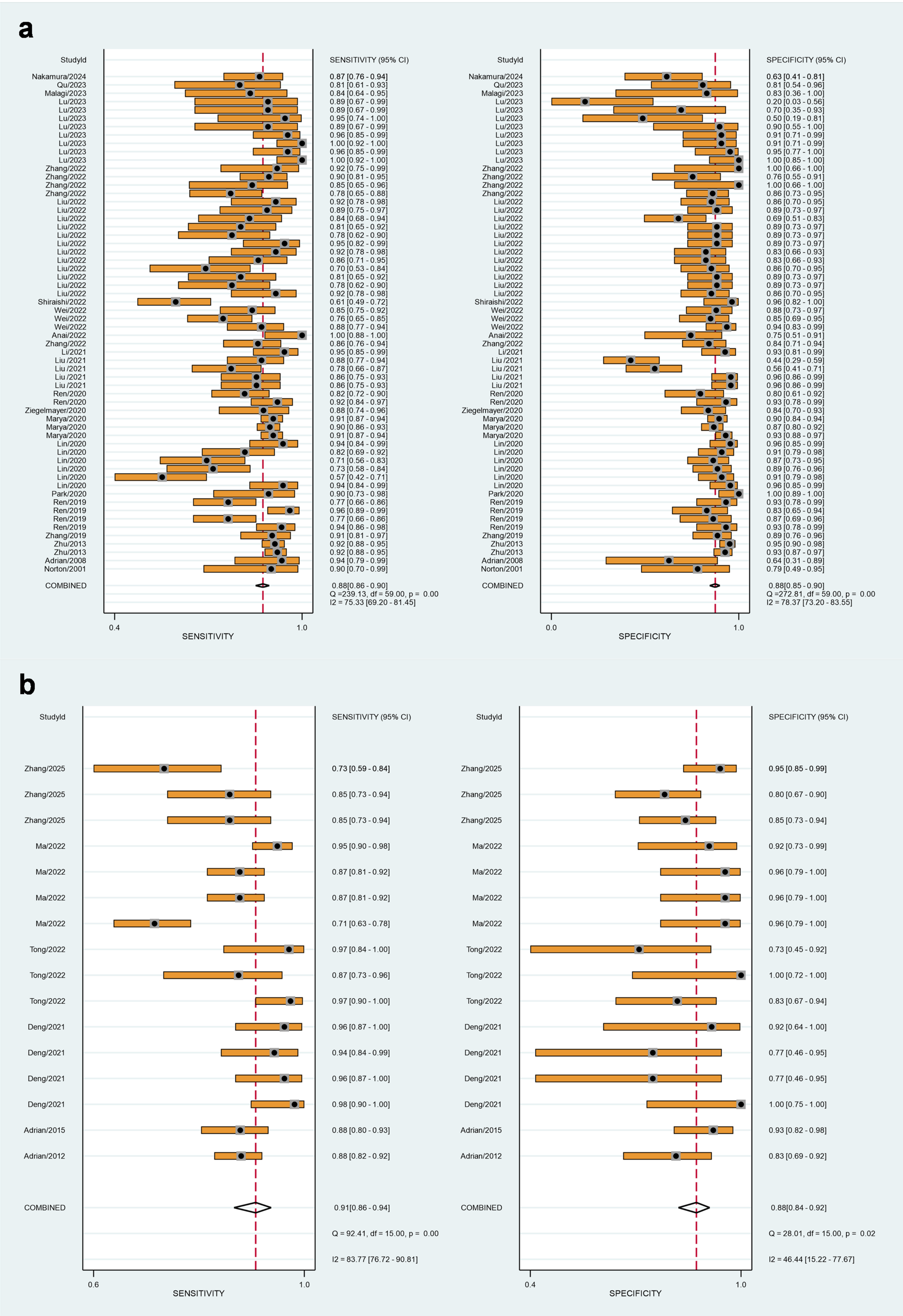


**Fig S16: Forest plot of different centers (Single or multiple).**

**a:** single (22 studies with 60 tables)

**b:** multi (6 studies with 16 tables)

**
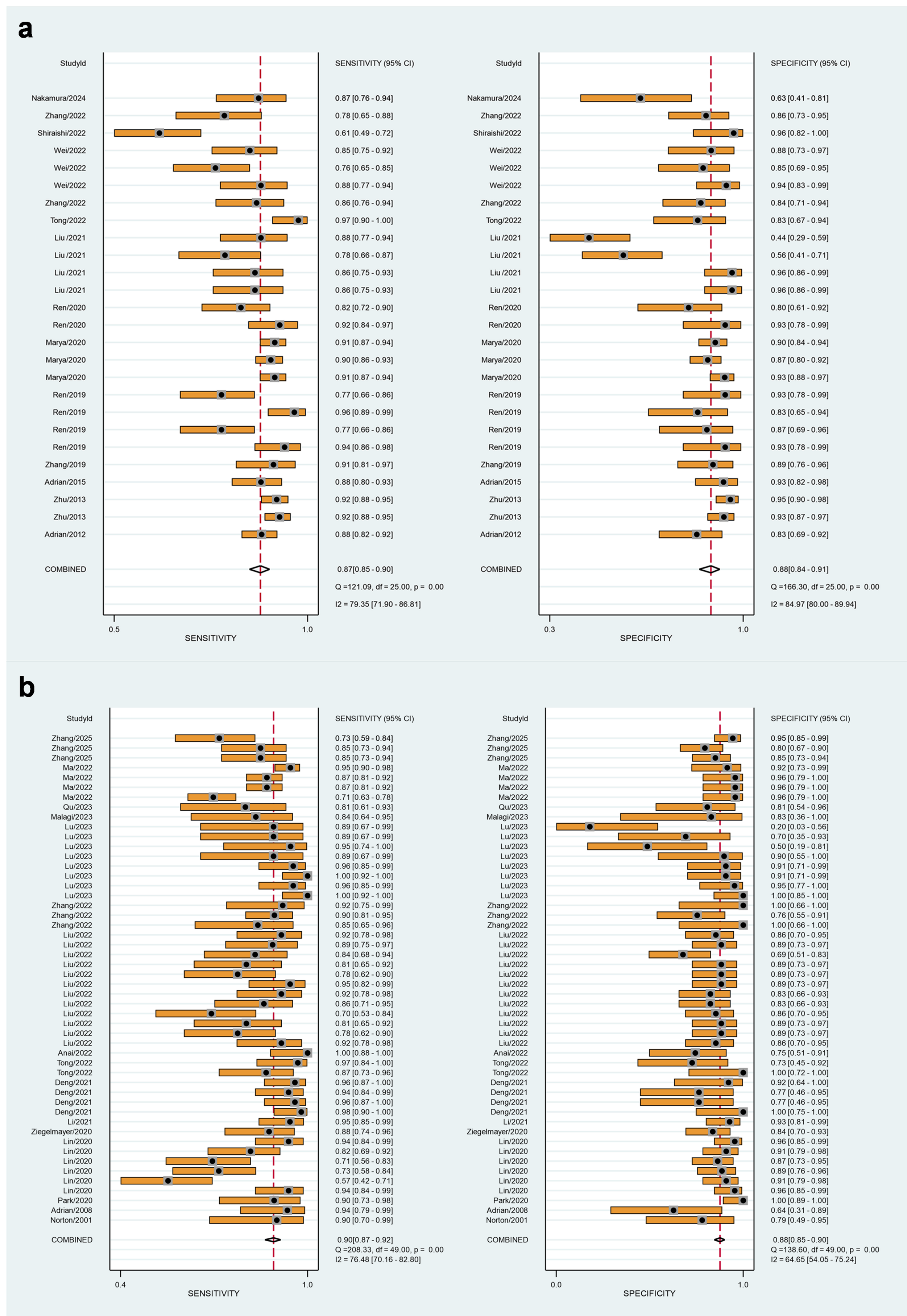
**

**Fig S17: Forest plot of different sample sizes (≤ 100 or > 100).**

**a:** Sample size ≤ 100 (14 studies with 26 tables)

**b:** Sample size > 100 (14 studies with 50 tables)

**
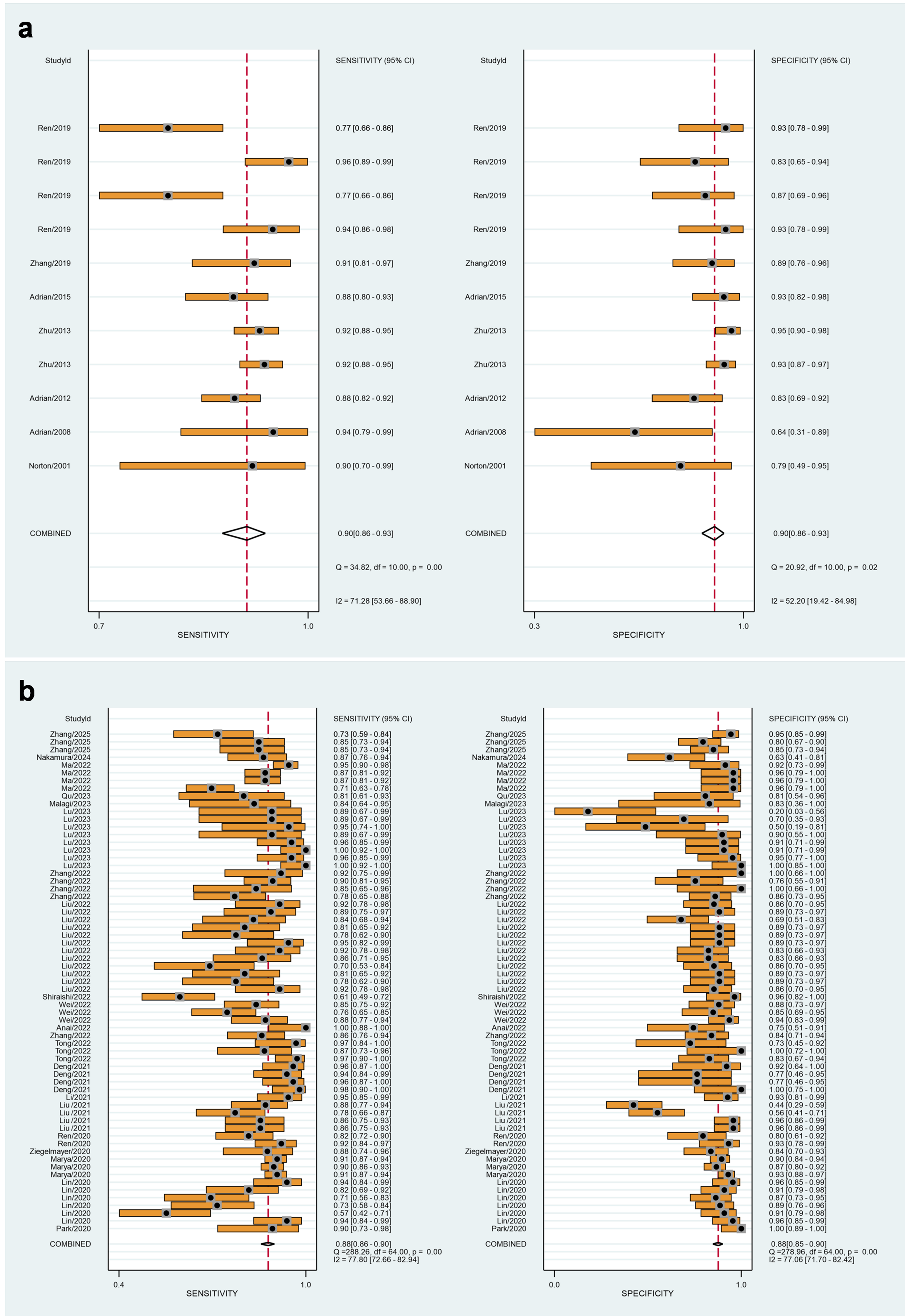
**

**Fig S18: Forest plot of different publication year (before or after 2020).**

**a:** Year of publication before 2020 (7 studies with 11 tables)

**b:** Year of publication after 2020 (21 studies with 65 tables)

**
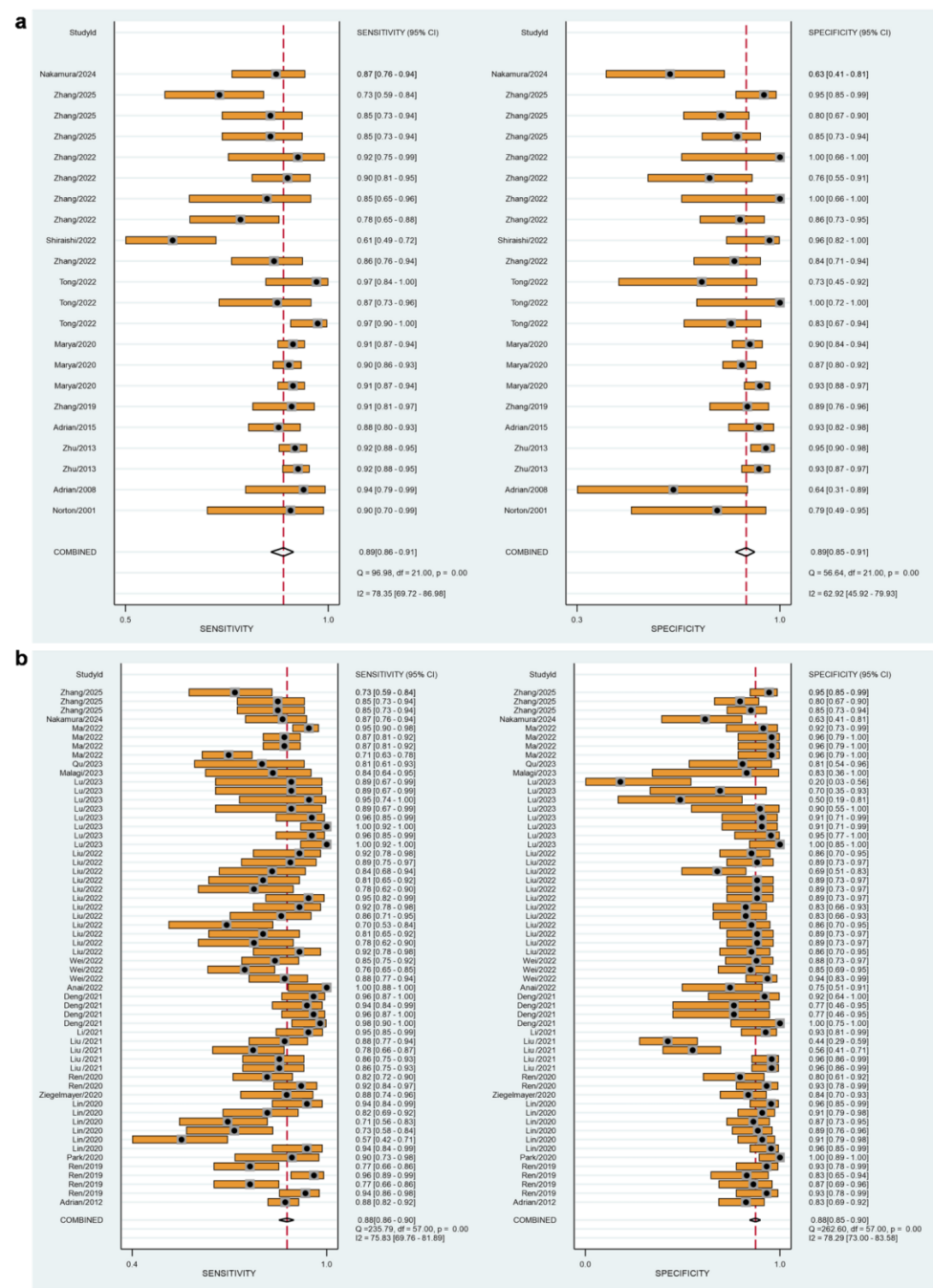
**

**Fig S19: Forest plot of different risk of bias levels (High/Unclear or Low).**

**a:** Low risk of bias and concern of applicability of study (9 studies with 30 tables)

**b:** High/Unclear risk of bias and concern of applicability of study (19 studies with 46 tables)


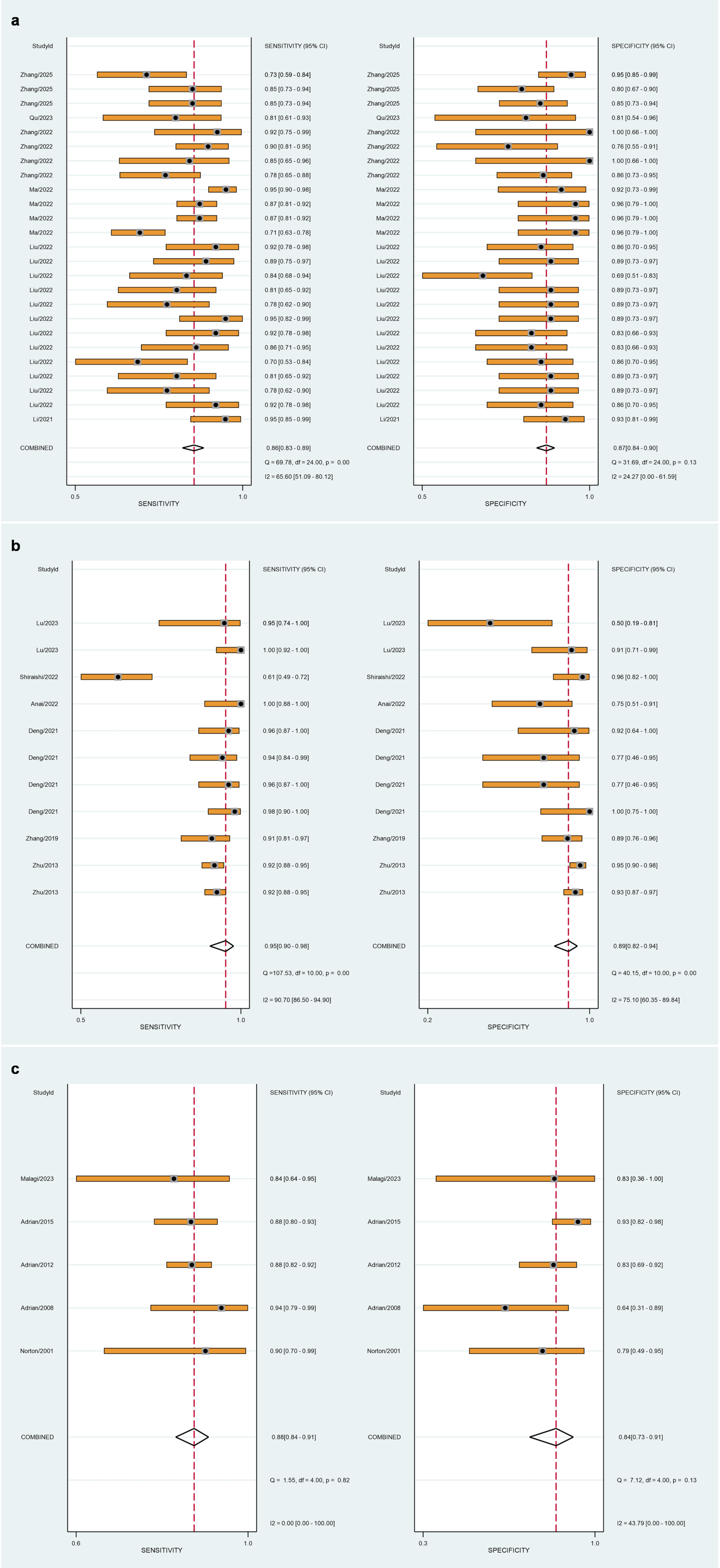


**Fig S20: Forest plot** of different AI algorithm**.**

**a:** Pooled AI algorithm of LASSO in this meta study. (6 studies with 25 tables)

**b:** Pooled AI algorithm of SVM in this meta study. (6 studies with 11 tables)

**c:** Pooled AI algorithm of ANNs in this meta study. (5 studies with 5 tables)
